# Novel 1,3-Diazepines as Nontoxic Corrosion Inhibitors

**DOI:** 10.1021/acsomega.5c07989

**Published:** 2025-10-30

**Authors:** Ana J. F. Souza, Priscila M. Souza, Gabriel R. Antunes, Maxwel E. Bille, Odeydes J. R. P. Carvalho, Alessandro D. Oliveira, Cecília S. Santos, Gabriela F. M. Lopes, Silmara N. Andrade, Fernando P. Varotti, Julliane Yoneda, Elivelton A. Ferreira, Diego Pereira Sangi

**Affiliations:** † 28110 Universidade Federal Fluminense, Instituto de Ciência Exatas, Departamento de Química, Rua Ellis Hermydio Figueira, 783, Aterrado, Volta Redonda, Rio de Janeiro 27213-145, Brazil; ‡ Companhia Siderúrgica Nacional (CSN), BR-393, Km 5001, Vila Santa Cecília, Volta Redonda, Rio de Janeiro 27264 120, Brazil; § Centro de Ciências da Saúde, 74383 Universidade Federal de São João Del-Rei, Praça Frei Orlando, 170, Centro, São João del Rei, Divinópolis, Minas Gerais 36307-352, Brazil

## Abstract

Nitrogen-containing heterocyclic compounds are among the most effective corrosion inhibitors, primarily acting through adsorption by donating electron pairs to metallic surfaces. While benzodiazepines and other 1,4- and 1,5-diazepine derivatives have demonstrated inhibitory activity, 1,3-diazepan-2-ylidenes remain unexplored in the literature. In the present study, ketene dithioacetals were employed as building blocks for the synthesis of a novel series of 2-substituted 1,3-diazepines. Their corrosion inhibition efficiency was systematically evaluated, alongside in silico predictions of toxicity risks and in vitro cytotoxicity assays against the MDA-MB-231 human breast adenocarcinoma cell line, the A549 human lung carcinoma cell line, the TOV-21G human ovarian adenocarcinoma cell line, and the WI-26VA4 human lung fibroblast cell line. The synthesized compounds exhibited significant corrosion inhibition performance, while in silico analyses indicated no relevant toxicity risks, findings further supported by low cytotoxicity observed in in vitro assays. These results highlight 2-substituted 1,3-diazepines as promising candidates for application as organic corrosion inhibitors.

## Graphical abstract

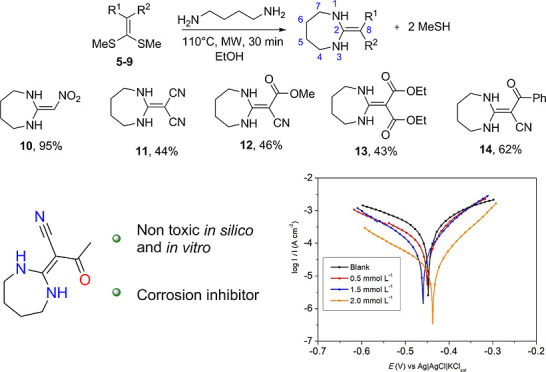

## 1 Introduction

Steel structures are highly susceptible to corrosion, a process that can lead to severe structural damage. This phenomenon may result in serious consequences, including occupational hazards, significant economic losses, and environmental contamination, primarily due to its potential to cause pipeline ruptures, equipment failures, and chemical leakage.^1–3^

One of the most effective strategies for mitigating corrosion is the use of inhibitors. Inorganic compounds such as chromates, phosphates, and molybdates have traditionally been employed due to their well-established efficiency in corrosion prevention. However, their application presents significant drawbacks, as these inhibitors are associated with environmental and health concerns.^3,4^

Alternatively, organic inhibitors offer several advantages, including biodegradability and low toxicity, which have stimulated extensive research in this field. Among them, the most efficient inhibitors are heterocyclic compounds containing oxygen and/or nitrogen atoms, which typically act through adsorption by donating electron pairs to metal surfaces.^3–8^

Diazepines represent a remarkable class of heterocyclic compounds owing to their broad range of applications in the biological sciences. Benzodiazepines were the first heterocycles to be recognized as privileged structures,^9,10^ and several derivatives, including 1,4-diazepines, 1,2-diazepines, and 1,5-diazepines, have also demonstrated noteworthy biological activities.^11–17^ More recently, studies have revealed that benzodiazepines, as well as other 1,4- and 1,5-diazepine derivatives, can act as effective corrosion inhibitors, even under strongly acidic conditions.^18–24^

The 1,3-diazepine scaffold is likewise considered a privileged structure in medicinal chemistry.^25^ This moiety occurs in a wide range of biologically active compounds, including several natural products.^26–30^ Although the synthesis of 1,3-diazepines has been extensively reported, 1,3-diazepan-2-ylidenes remain virtually unexplored, making their synthesis and the investigation of their properties a particularly appealing area of research.^31,32^

Ketene dithioacetals are valuable building blocks in organic synthesis, particularly due to their ability to undergo double vinylic substitution, enabling the formation of five- and six-membered heterocycles with relevant biological activities.^33–35^ In recent studies, we have also investigated 2-nitromethylene oxazolidine (**1**), imidazolidine (**2**), oxazinane (**3**), and hexahydropyrimidine (**4**), all derived from 1,1-bis­(methylsulfanyl)-2-nitroethylene (**5**), as potential corrosion inhibitors (Figure 1).^36–40^

**Figure 1:**
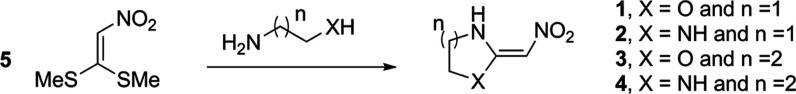
2-Nitromethylene heterocycles corrosion inhibitors.

In this work, ketene dithioacetals were employed for the synthesis of novel 1,3-diazepine derivatives, whose potential application as corrosion inhibitors for the protection of carbon steel was investigated.

## 2 Materials and Methods

### 2.1 General Informations

All chemicals were purchased from commercial suppliers and used without further purification. Ketene dithioacetals (**5–9**) were synthesized via a three-step, one-pot procedure, as previously described by Baliza et al.^37^

Microwave-assisted reactions were performed using an Anton Paar Monowave 300 device. Thin-layer chromatography analyses were conducted on commercial aluminum plates coated with 0.2 mm silica gel (Macherey-Nagel), and compounds were visualized under ultraviolet light at 254 nm.

Infrared spectra were recorded on a Bruker FT-IR Vertex 70 spectrophotometer using the attenuated total reflectance (ATR) mode. Nuclear magnetic resonance (NMR) analyses were performed on a Bruker Avance DRX 400 MHz and a Varian VNMRS 500 MHz spectrometer. Mass spectrometry (MS) analyses were carried out using a Shimadzu GCMS-QP2010 Plus and a Waters/Micromass UPLC-QTof-MS system, providing a resolution exceeding 10,000 fwhm.

### 2.2 General Procedure for the Synthesis of 1,3-Diazepines

In a microwave-compatible glass vessel, the polarized dithioacetal (**5–9**) (1 mmol) and 1,4-diaminobutane (1 mmol) were dissolved in ethanol (3 mL). The reaction mixture was subjected to microwave irradiation for 60 min at 110 °C with continuous stirring. Upon completion, the solvent was evaporated, and the resulting products were obtained in pure form following purification by column chromatography using a dichloromethane/ethyl acetate mixture as the eluent.

The data from the infrared spectroscopy, mass spectrometry and nuclear magnetic resonance spectra of hydrogen (^1^H NMR) and carbon (^13^C NMR) used for compound identification are presented as follows.

2-(nitromethylene)-1,3-diazepine (**10**): Yield 95%. ^1^H NMR (500 MHz, DMSO): δ 8.83 (s, 2*H*), 6.39 (s, 1*H*), 3.71–3.52-(m, 4*H*), 1.71–1.65 (m, 4*H*); ^13^C NMR (126 MHz, DMSO): δ 162.81, 100.49, 44.62, 27.42. IR (ATR) (*v*_max_/cm^–1^): 3292, 1594, 1353, 1189, 983, 763. MS (*m*/*z*, (%)): 157(58); 123(42); 70(63); 55(94); 44(100) Calcd for C_6_H_12_N_3_O_2_^+^ [M + H]^+^ = 158.0931; found, 158.0954.

2-(1,3-Diazepan-2-ylidene)­malononitrile (**11**): Yield 44%. ^1^H NMR (400 MHz, DMSO): δ 7.62 (s, 2*H*), 3.18–3.12 (m, 2*H*), 1.56–1.48 (m, 2*H*). ^13^C NMR (101 MHz, DMSO): δ 168.26, 119.06, 45.55, 33.89, 27.44. IR (ATR) (*v*_max_/cm^–1^): 3288, 2221, 2173, 1573, 1363, 709. MS (*m*/*z*, (%)): 162(100); 133(37); 44(72). Calcd for C_8_H_11_N_4_^+^ [M + H]^+^ = 163.0985; found, 163.0982.

Methyl 2-cyano-2-(1,3-diazepan-2-ylidene)­acetate (**12**): Yield 46%. ^1^H NMR (400 MHz, DMSO): δ 8.10–7.87 (m, 2H), 3.57 (s, 3H), 3.31–3.25 (m, 4*H*), 1.67–1.56 (m, 4*H*). ^13^C NMR (101 MHz, DMSO): δ 168.94, 167.91, 119.55, 55.96, 50.43, 44.64, 26.93. IR (ATR) (*v*_max_/cm^–1^): 3294, 2188, 1635, 1612, 1282, 1128, 671. MS (*m*/*z*, (%)): 195(100); 164(92); 112(40). HRMS (ESI) Calcd for C_9_H_14_N_3_O_2_^+^ [M + H]^+^ = 196.1079; found, 196.1089.

Diethyl 2-(1,3-diazepan-2-ylidene)­malonate (**13**): Yield 43%. ^1^H NMR (400 MHz, MeOD): δ 4.09 (q, *J* = 7.1 Hz, 4*H*), 3.26–3.21 (m, 4*H*), 1.72–1.65 (m, 4*H*), 1.25 (t, *J* = 7.1 Hz, 6*H*). ^13^C NMR (101 MHz, MeOD): δ 171.22, 170.54, 76.64, 59.08, 45.14, 27.70, 13.37. IR (ATR) (*v*_max_/cm^–1^): 3289, 1606, 1141, 1070, 792. HRMS (ESI) Calcd for C_12_H_21_N_2_O_4_^+^ [M + H]^+^ = 257.1502; found, 257.1510.

2-(1,3-Diazepan-2-ylidene)-3-oxo-3-phenylpropanenitrile (**14**): Yield 62%. ^1^H NMR (400 MHz, CDCl_3_): δ 11.35 (s, 1H), 7.80–7.70 (m, 2*H*), 7.50–7.38 (m, 3*H*), 5.86 (s, 1*H*), 3.45 (s, 4*H*), 1.86 (s, 4*H*). ^13^C NMR (101 MHz, CDCl_3_): δ 190.45, 168.80, 140.13, 130.69, 127.99, 127.72, 122.27, 70.91, 45.31, 27.32. IR (ATR) (*v*_max_/cm^–1^): 3278, 2187, 1599, 1347, 702. MS (*m*/*z*, (%)): 241(100); 240(69); 105(42); 77(70) Calcd for C_14_H_16_N_3_O^+^ [M + H]^+^ = 242.1291; found, 242.1233.

### 2.3 Calculation of Theoretical Parameters

Certain theoretical parameters calculated using density functional theory (DFT) can be correlated with the inhibition efficiency of a compound.^41^ The structures of the studied compounds were optimized at the B3LYP/6–311G++(d,p) level of theory using the Gaussian 16W program^42^ to obtain these parameters.

The energies of Frontier orbitals (*E*_HOMO_ and *E*_LUMO_) were calculated from the optimized geometries and are related to the ionization potential (I) and electron affinity (A) as described in eqs 1 and 2.^41^

1 $$I=-E_{HOMO}$$

2 $$A=-E_{LUMO}$$

Additional physicochemical parameters related to inhibition efficiency, such as chemical hardness (η) and softness (σ) were calculated from the ionization energy and electron affinity according to eqs 3 and 4.^41^

3 η=(I−A)/2

4 σ=1/η

### 2.4 Procedure of Mass Loss Evaluation

SAE 1020 steel plates, with a composition of C (0.18–0.23%), Mn (0.3–0.6%), P (0.03%), and S (0.05%), and dimensions of 2.5 × 2.5 × 0.13 cm, were polished sequentially using emery papers of increasing grit sizes (150, 220, 320, 400 and 600). After polishing, the plates were thoroughly rinsed with deionized (Milli-Q) water.

A control solution was prepared by dissolving 5% (v/v) dimethyl sulfoxide in HCl 1 mol L^–1^. Inhibitor-containing solutions were prepared by adding 2 mmol L^–1^ of the inhibitor to 5% (v/v) dimethyl sulfoxide in HCl 1 mol L^–1^ using deionized (Milli-Q) water. SAE 1020 steel plates were immersed in these solutions for a duration of 4 h.

The gravimetric method was employed to evaluate corrosion resistance by weighing the steel plates before and after immersion in the aqueous HCl solution. The mass loss (w) was calculated as the difference between the average initial mass (*m*_i_) and final mass (*m*_f_) of each plate (eq 5). This information was subsequently used to determine the inhibition efficiency (IE) according to eq 6, where *w*_0_ and *w* represent the mass losses in the absence and presence of the inhibitor, respectively. All measurements were performed in triplicate to ensure accuracy and reliability, and the reported values correspond to the average of these three determinations.^39,40^

5 $$w=m_{f}-m_{i}$$

6 $$IE\;\left(\%\right)=\frac{\left(w^{0}-w\right)}{w^{0}}.100$$

### 2.5 Electrochemical Measurements

The aqueous solutions were prepared from 5% (v/v) dimethyl sulfoxide in HCl 1 mol L^–1^ in the absence and presence of inhibitors with inhibitor concentrations of 0.5, 1.5, and 2.0 mmol L^–1^. The SAE 1020 steel was immersed in acid solutions for 30 min at 25 °C in open-circuit potential (OCP) conditions. Afterward, polarization curve (PC) analyses were performed, using an EmStat3+ potentiostat from PalmSens. A minimum of two runs were conducted for each experiment. An initial potential in the cathodic region (−150 mV vs OCP) was applied to measure the PC, with a sweep rate of 0.166 mV s^–1^ toward more positive potentials until reaching +150 mV vs OCP.^38,39^

### 2.6 SEM and EDS Analysis

Scanning electron microscopy (SEM) and energy dispersive Spec-troscopy (EDS) analysis were acquired using an FEI Quanta 3D FEG instrument.^39^

### 2.7 In Silico Toxicity Evaluation

The toxicity risks of the studied compounds were assessed in silico using the OSIRIS Property Explorer server.^43^ To corroborate these results, Toxtree was employed to estimate potential toxic hazards.^44^ The Benigni/Bossa rulebase for mutagenicity and carcinogenicity, a module within Toxtree, was applied.^45^

### 2.8 Procedure to Cytotoxicity Evaluation

#### 2.8.1 Cell Culture

The MDA-MB-231 human breast adenocarcinoma cell line (ATCC HTB-26), A549 human lung carcinoma cell line (ATCC CCL-185), TOV-21G human ovarian adenocarcinoma cell line (ATCC CRL-11730), and WI-26VA4 human lung fibroblast cell line (ATCC CCL-95.1) were cultured in RPMI-1640 medium supplemented with 10% fetal bovine serum (FBS) and gentamicin (100 μg/mL). Cells were maintained at 37 °C in a humidified atmosphere containing 5% CO_2_.^46^

#### 2.8.2 Assessment of Cell Viability by MTT Assay

Cell viability was assessed using the MTT assay (3-[4,5-dimethylthiazol-2-yl]-2,5-diphenyl-tetrazolium bromide). Briefly, 100 μL of complete medium containing 1 × 10^4^ cells was added to each well of a 96-well tissue culture plate. Cells were incubated at 37 °C in a humidified atmosphere with 5% CO_2_ for 24 h prior to treatment. Following medium removal, 100 μL of fresh medium containing the test compounds at concentrations ranging from 0.01 to 100 μM was added to each well, and cells were incubated for 48 h under the same conditions. All compounds were presolubilized in dimethyl sulfoxide (DMSO), and the final DMSO concentration in all treatments was maintained at ≤ 0.2% to avoid interference with cell viability.

After treatment, the medium was replaced with 100 μL of MTT solution (0.5 mg/mL) per well, followed by a 3 h incubation. Formazan crystals were dissolved by adding 100 μL of DMSO to each well, and absorbance was measured at 550 nm using a microplate reader (SpectraMax M5e, Molecular Devices, Sunnyvale, CA, USA). The percentage of growth inhibition was calculated as [1-(Abs of treated/Abs of control)]×100. All experiments were conducted in triplicate, and results are expressed as mean IC_50_ values, which were determined using OriginPro 8.0 software (OriginLab Corporation, Northampton, MA, USA).^37^

## 3 Results and Discussion

Using ketene dithioacetals (**5–9**), a microwave-assisted synthesis was performed with 1,4-diaminobutane as the nucleophile to obtain 2-substituted 1,3-diazepines. Their corrosion inhibition properties were subsequently evaluated to assess the potential application of these compounds in the protection of carbon steel (Figure 2). Five 2-substituted 1,3-diazepines (**10–14**) were successfully synthesized, with yields ranging from 43 to 95%.^35–37^

**Figure 2:**
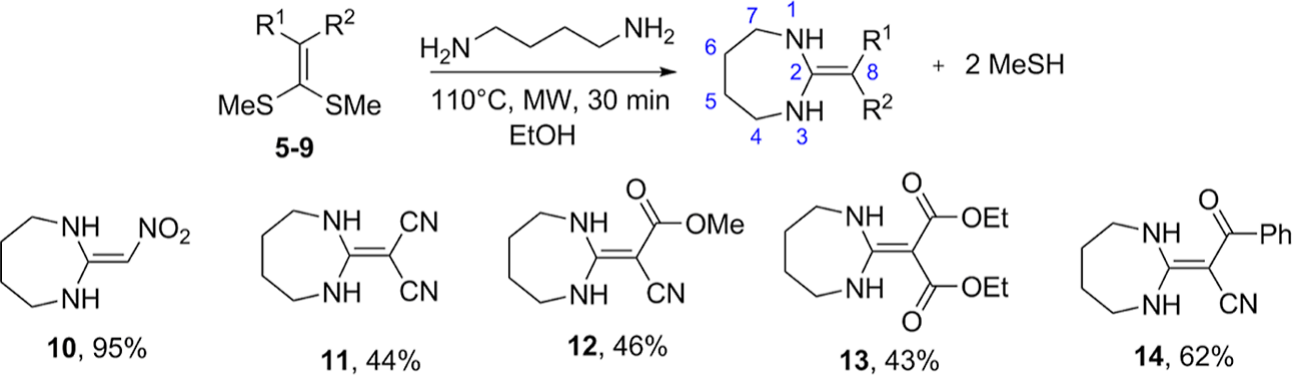
Synthesis of 1,3-diazepines (**10–14**).

Analysis of the ^1^H NMR spectra indicated that derivatives **10–14** did not display the singlets in the 2.5–2.8 ppm range, which are characteristic of the methylsulfanyl groups present in ketene dithioacetals (**5–9**). As anticipated, compounds **10–14** exhibited signals between 3.1 and 3.7 ppm, corresponding to 4H from CH_2_ groups bonded to N–H, as well as signals between 1.4 and 1.7 ppm, also integrating to 4H, assigned to the methylene groups at positions 5 and 6, completing the diazepine ring.

In the ^13^C NMR spectra of compounds **10–14**, signals were observed at 27 ppm for carbons 5 and 6, and at 45 ppm for carbons 4 and 7. Notably, due to the mesomeric effect of the nitrogen lone pair and the resonance of the exocyclic double bond with electron-withdrawing substituents, the signals corresponding to the sp^2^ carbons 2 and 8 are significantly shifted, with carbon 2 appearing in the range of 34–100 ppm and carbon 8 in the range of 162–170 ppm. Complete signal assignments, including those for the R^1^ and R^2^ substituents, are provided in the Supporting Information.

Calculated physicochemical parameters for the studied compounds are summarized in Table 1.

**Table 1:** Physicochemical Parameters Calculated for Compounds **10**–**14**

compound	*E*_HOMO_ (eV)	*E*_LUMO_ (eV)	Δ*E* (eV)	η	σ
**10**	–6.35	–1.75	4.60	2.30	0.44
**11**	–6.65	–1.72	4.94	2.47	0.41
**12**	–6.18	–1.01	5.16	2.58	0.39
**13**	–5.95	–0.61	**5.33**	**2.67**	**0.37**
**14**	–6.23	–1.66	**4.57**	**2.29**	**0.44**

Although the calculated data offer valuable insights into the behavior of the inhibitors, a direct correlation between inhibition efficiency and physicochemical parameters could not be established, as the substituents at positions R^1^ and R^2^ vary simultaneously. Nonetheless, the analysis enabled the identification of the most and least promising compounds in terms of inhibitory potential.

The energy gap (Δ*E*), defined as the difference between the energies of the Frontier molecular orbitals, is closely associated with molecular stability and reactivity. In general, a larger Δ*E* implies lower chemical reactivity and, consequently, reduced inhibition efficiency.^47^ According to the data presented in Table 1, compound **13** exhibits the highest Δ*E* among the analyzed molecules, suggesting it may act as the least effective corrosion inhibitor. In contrast, compound **14**, with the lowest Δ*E*, is expected to demonstrate superior inhibitory performance.

Considering the delocalized and extensive electron cloud characteristic of metallic surfaces, these are typically classified as “soft” in the context of chemical reactivity. Accordingly, “soft” molecules tend to interact more effectively with such surfaces and are therefore considered better corrosion inhibitors.^47^ In this context, the analysis of the global hardness and softness parameters calculated for the studied compounds (Table 1) suggests that compound **13** is likely to exhibit the lowest inhibition efficiency, whereas compound **14** presents the most favorable profile and is expected to act as the most effective inhibitor.

Carbon steel samples were weighed and exposed to a corrosive environment consisting of an HCl solution, both in the absence and presence of diazepine compounds. After a 4 h exposure under conditions favorable for metal oxidation, it was observed that all tested diazepines exhibited corrosion inhibition activity. The 1,3-diazepines demonstrated excellent inhibition efficiency in mass loss experiments, with values ranging from 88% to 94% at a concentration of 2 mM, with the exception of compound **13**, which showed significantly lower performance, inhibiting only 19% under the same conditions (Table 2).

**Table 2:** Corrosion Inhibition Efficiency Determined by Mass Loss Tests

inhibitor	IE (%)^a^ ^,^ ^b^
**10**	91 ± 0.7
**11**	88 ± 0.9
**12**	92 ± 0.6
**13**	19 ± 5.4
**14**	94 ± 2.5

a Relative to mass loss of the solution (5% v/v in HCl 1M) without inhibitor.

b Standard deviations on percentage of mass.

The experimental results are consistent with the physicochemical descriptors calculated and presented in Table 1. Although a direct quantitative correlation between the theoretical parameters and the observed inhibition efficiencies cannot be firmly established, the data support the conclusion that compound **13** is likely to exhibit the lowest inhibition performance among the studied compounds, whereas compound **14** is expected to be the most effective.

The poor inhibition performance of compound **13** is likely associated with the presence of intramolecular hydrogen bonding (NH···O=C distance of 1.78 Å), which, unlike in the other compounds analyzed, occurs on both sides of the molecule. This intramolecular interaction may reduce the availability of electron lone pairs by stabilizing them through internal bonding, thereby making them less accessible for interaction with the metal surface and diminishing the compound’s ability to inhibit corrosion effectively.

For the inhibitor with the best efficiency (**14**), electrochemical tests were also carried out at different concentrations. Figure 3 shows the PC for the SAE 1020 steel in 0.1 mol L^–1^ HCl solution, in the absence and presence of compound **14**. For solutions with 0.5 and 1.5 mmol L^–1^ of inhibitor, there was a decrease in the current density values of the cathodic branch only. However, at 2.0 mmol L^–1^, both the current densities of the cathodic and anodic branches showed significant decreases, showing consistency with the mass loss results. It can also be observed that the OCP shift is smaller than 85 mV, indicating a mixed-type inhibitor.

**Figure 3:**
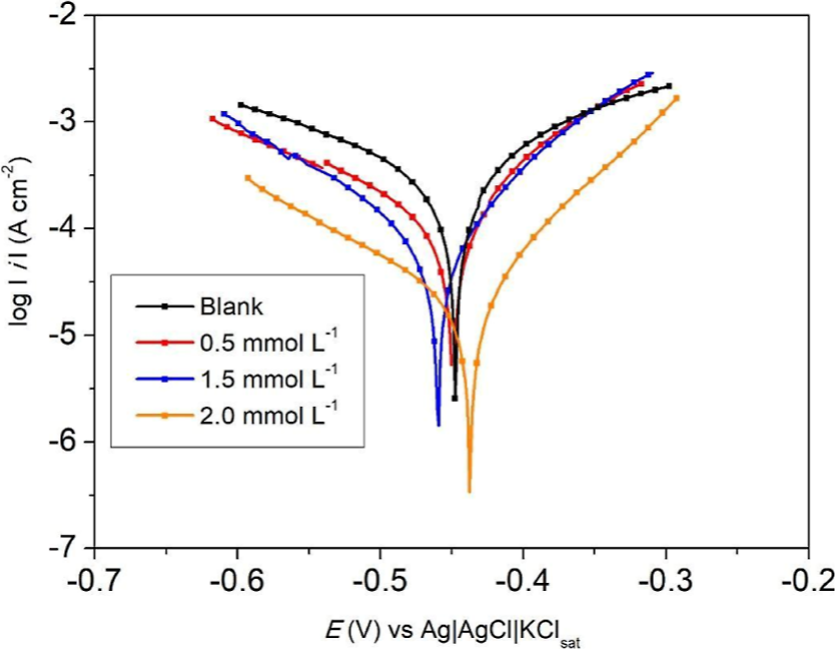
PC for the SAE 1020 steel in 0.1 mol L^–1^ HCl solution, in the absence and presence of different concentrations of compound **14**.

Figure 4. SEM micrographs and EDS results of the SAE 1020 steel and the samples immersed for 4 h in HCl solution, in the absence (a) and presence of the 12 (b), and 14 (c) inhibitors at 2.0 mmol L^–1^.

**Figure 4:**
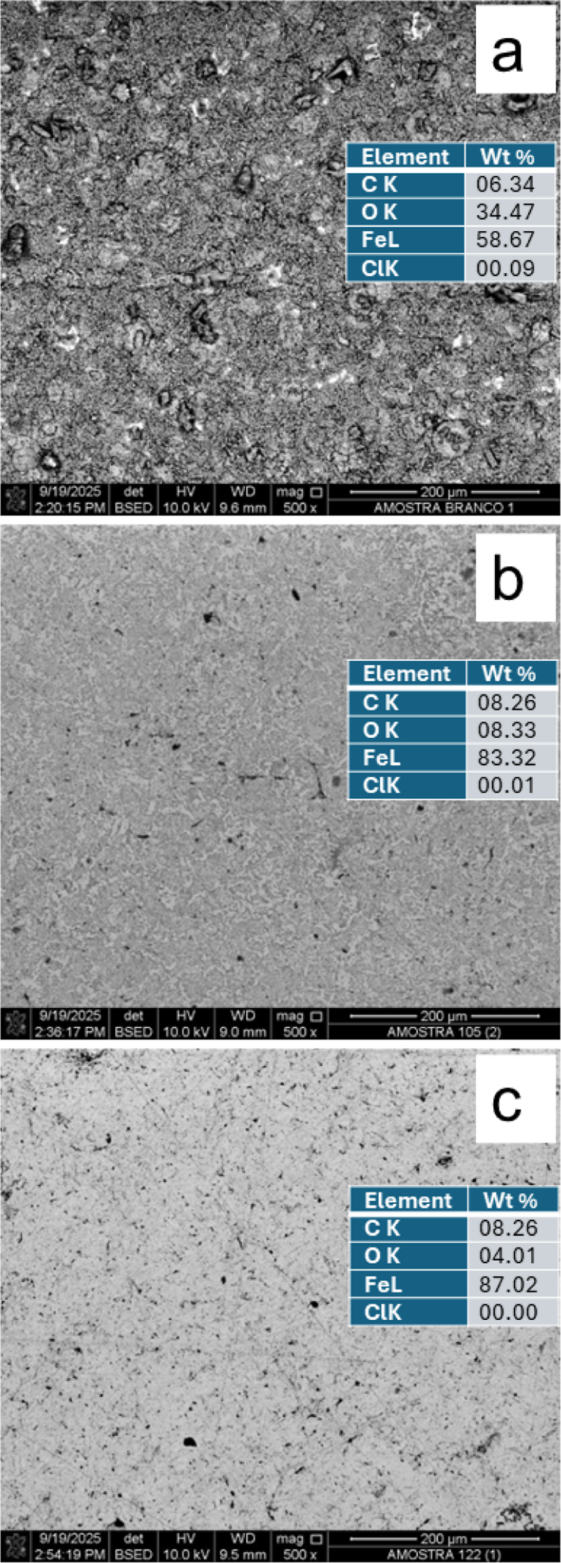
Presents SEM micrographs and EDS results of the SAE 1020 steel and the samples immersed for 4 h in HCl solution, in the absence and presence of the inhibitors 12 and 14 at 2.0 mmol L^–1^.

The SEM micrograph of the sample immersed in a solution without inhibitor (Figure 4a) shows the presence of corrosion products possibly associated with the oxidation of the steel after removal from the acidic solution and exposure to the atmosphere, since there were no corrosion inhibitors in the solution that could adsorb on the surface of the sample, ensuring protection after removal from the solution as well.

On the other hand, the steel samples immersed in solution with the presence of inhibitor 14 presented smaller amounts of corrosion products in relation to the sample exposed to inhibitor 12 (Figure 4b,c), showing that these results are also consistent with those of mass loss and PC.

Elemental quantification of corrosion products by EDS confirmed the observations made in the SEM micrograph. The sample immersed in solution with inhibitor 14 had the lowest oxygen content (4.01 wt %), followed by the samples exposed to the solution containing inhibitor 12 (8.33 wt %) and the sample immersed in a solution without inhibitors (34.47 wt %). The sample immersed in the solution with inhibitors had lower Fe contents associated with its oxidation by atmospheric oxygen. Chloride traces were also detected on the surface of this sample and on the sample immersed in the solution with inhibitor 12. No chloride was detected in the sample immersed in the solution with inhibitor 14.

Regarding toxicity risks, the results obtained from OSIRIS Property Explorer indicate that compounds **10–14** do not exhibit mutagenic, tumorigenic, irritant, or reproductive toxicity risks (Figure 5), with the exception of compound **12,** which shows a potential irritant effect.

**Figure 5:**
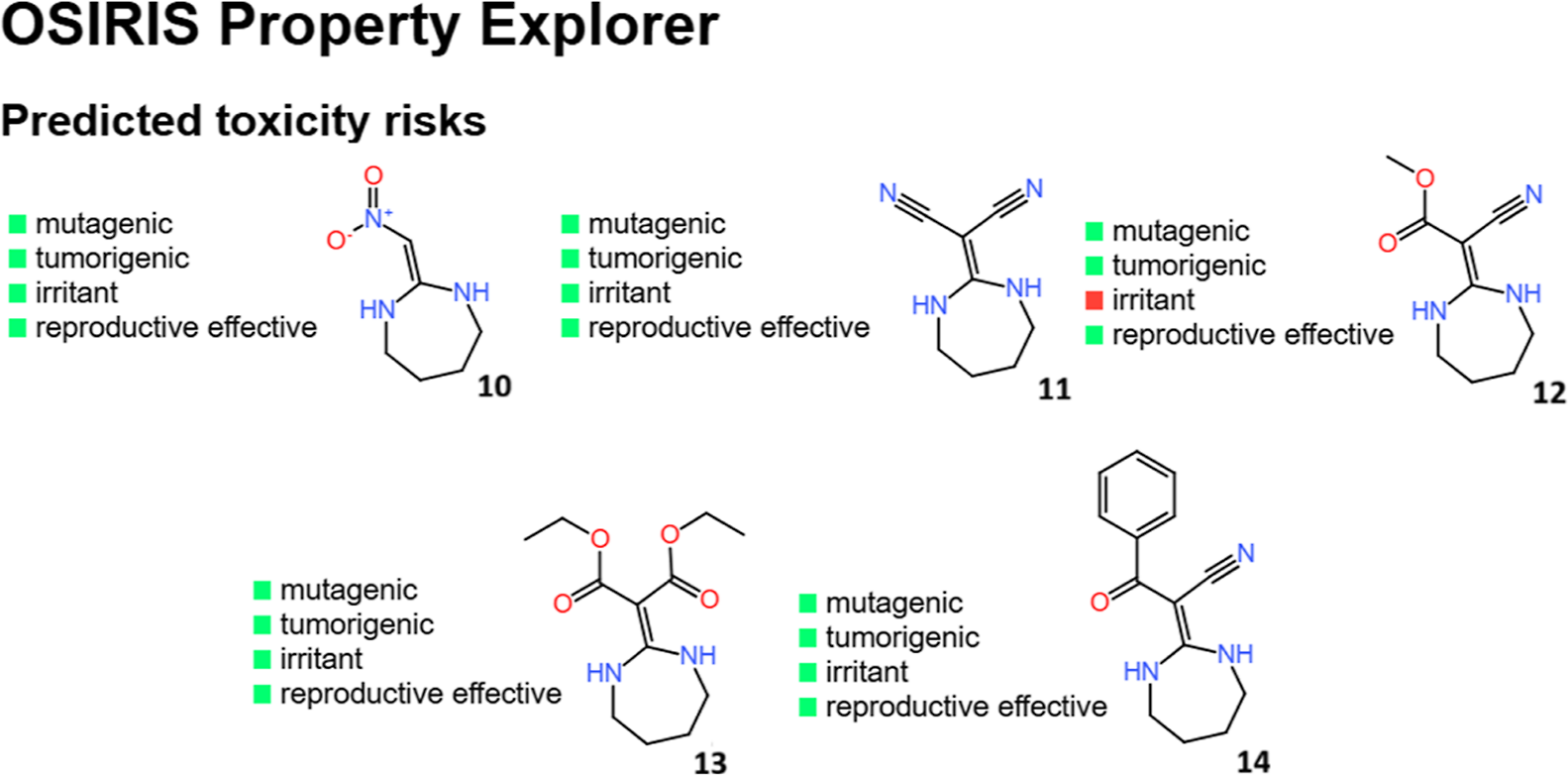
Toxicity risks predicted by the OSIRIS Property Explorer server for compounds **10–14** proposed as nontoxic corrosion inhibitors.

The Toxtree assessment yielded negative results for both genotoxic and nongenotoxic carcinogenicity for the compounds studied. Additionally, no structural alerts for *Salmonella typhimurium* mutagenicity were detected, except for compound **14**, for which the program identified structural alerts related to *S. typhimurium* mutagenicity as well as genotoxic carcinogenicity.

The discrepancies observed between the toxicity predictions from OSIRIS and Toxtree are attributable to the different underlying models employed by each tool. OSIRIS utilizes the registry of toxic effects of chemical substances (RTECS) database (Actelion Pharmaceuticals Ltd., USA) for toxicity prediction, whereas Toxtree applies a decision tree-based approach to estimate toxic hazards. Despite these methodological differences, both tools consistently indicate that the majority of the studied compounds present no significant toxicity risks. These in silico predictions serve as an initial screening method, providing a rapid assessment of the toxicological profile and enabling the conservation of time and resources, but does not replace experimental evaluations. Based on these findings, a further experimental evaluation of the compounds’ toxicity would be warranted.

To evaluate the cytotoxic potential of the synthesized compounds, cell viability assays were performed using four human cell lines: MDA-MB-231, A549, TOV-21G, and WI-26VA4. The results, summarized in Table 3, indicate that none of the compounds exhibited significant cytotoxic activity, as evidenced by IC_50_ values exceeding 100 μM.

**Table 3:** In Vitro Cytotoxicity (IC_50_ Values) of the Synthesized Compounds and Doxorubicin Obtained against MDA-MB-231, A549, TOV-21G and WI-26VA4 Cell Lines

compounds	IC_50_ (μM) ± S.D^a^			
	MDA-MB-231	A549	TOV-21G	WI-26VA4
**10**	>100	>100	>100	>100
**11**	>100	>100	>100	>100
**12**	>100	>100	>100	>100
**13**	>100	>100	>100	>100
**14**	>100	NT^b^	>100	>100
**doxorubicin**	2.6 ± 0.8	0.67 ± 0.4	8.3 ± 4.0	7.0 ± 2.0

a S.D.: standard deviation.

b NT: not tested.

Analysis of the cell viability curves (Figure S32) revealed that increasing concentrations of the synthesized compounds did not elicit a dose-dependent response. Most of the tested cell lines maintained viability above 50% even at the highest concentration evaluated (100 μM).

In many in vitro cellular screening studies, compounds exhibiting IC_50_ values greater than 100 μM in standard viability assays, such as the MTT assay, are generally classified as noncytotoxic or as having low cytotoxic potential.^48^

In parallel, doxorubicin was employed as a positive control (Figure S33), exhibiting IC_50_ values in the expected low micromolar range across all tested cell lines,^49^ in stark contrast to the results obtained for the synthesized compounds.

The absence of toxicity risks in silico predictions, coupled with the lack of cytotoxicity in vitro and demonstrated inhibition efficiency, positions 1,3-diazepines **10**, **11**, **12** and **14** as promising candidates for safe and effective corrosion inhibitors.

## 4 Conclusions

Despite the extensive information available on the synthesis of diazepines and their applications as corrosion inhibitors, 1,3-diazepan-2-ylidenes remain virtually unexplored. To investigate the properties of these 1,3-diazepinic derivatives—both as corrosion inhibitors and in terms of cytotoxicity—we propose their synthesis via double vinylic substitution on ketene dithioacetals using 1,4-diaminobutane as the nucleophile.

Using this method, we synthesized five previously unreported 2-substituted 1,3-diazepines with yields ranging from 43% to 95%. Compounds **10**, **11**, **12** and **14** demonstrated corrosion inhibition efficiencies between 88% and 94% in mass loss tests conducted at an inhibitor concentration of 2 mM. The poor performance of compound **13** was related to its calculated theoretical parameters and to its intramolecular hydrogen bond on both sides of the molecule.

The majority of the studied compounds did not present toxicity risks according to in silico predictions, and none exhibited cytotoxic activity in vitro against human cell lines. These findings position the 1,3-diazepines **10**, **11**, **12** and **14** as promising candidates for the development of innovative, nontoxic organic corrosion inhibitors.

## Supplementary Material



## Notes

The authors provide in a separate file, additional experimental information as Infrared, mass and NMR spectra and cytotoxicity in different concentrations of the synthesized compounds (PDF)
