# Supplementary material for: Novel 1,3-Diazepines as Nontoxic Corrosion Inhibitors
Source: ACS Omega. 2025 Oct 30;10(44):53289–96. doi: 10.1021/acsomega.5c07989 (PMC12612871; doi:10.1021/acsomega.5c07989)

Supporting Information for

Novel 1,3-diazepines as non-toxic corrosion inhibitors

Ana J. F. Souza,[a] Priscila M. Souza,[a] Gabriel R. Antunes,[a] Maxwel E. Bille,[a] Odeydes J. R. P. Carvalho,[a] Alessandro D. Oliveira,[b] Cecília S. Santos,[c] Gabriela F. M. Lopes,[c] Silmara N. Andrade,[c] Fernando P. Varotti,[c] Julliane Yoneda,[a] Elivelton A. Ferreira,[a] Diego Pereira Sangi [a]*

[a] Departamento de Química, Instituto de Ciência Exatas, Universidade Federal Fluminense, Volta Redonda, Rio de Janeiro, Brazil.
[b] Companhia Siderúrgica Nacional (CSN), Volta Redonda, Rio de Janeiro, Brazil.
[c] Centro de Ciências da Saúde, Universidade Federal de São João Del-Rei, Divinópolis, Minas Gerais, Brazil

[*] Corresponding author: dpsangi@id.uff.br ; https://orcid.org/0000-0003-0388-5409.

Figure S1. ^{1}H NMR (500 MHz) spectrum of 2-(nitromethylene)-1,3-diazepine in DMSO- d_6 (**10**).

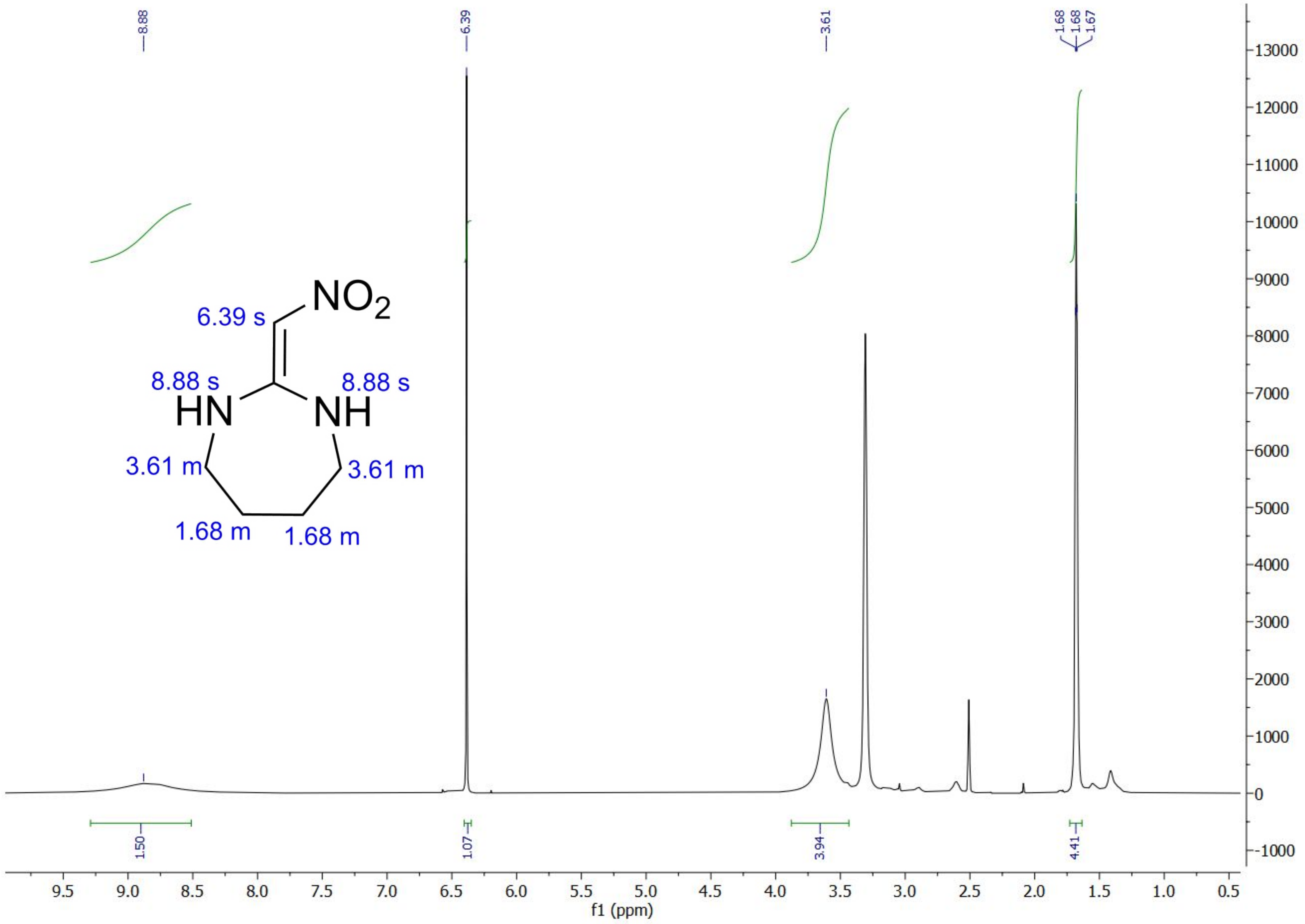

Figure S2. ^{1}H NMR (500 MHz) spectrum of 2-(nitromethylene)-1,3-diazepine in DMSO- d_6 expansion 1 (**10**).

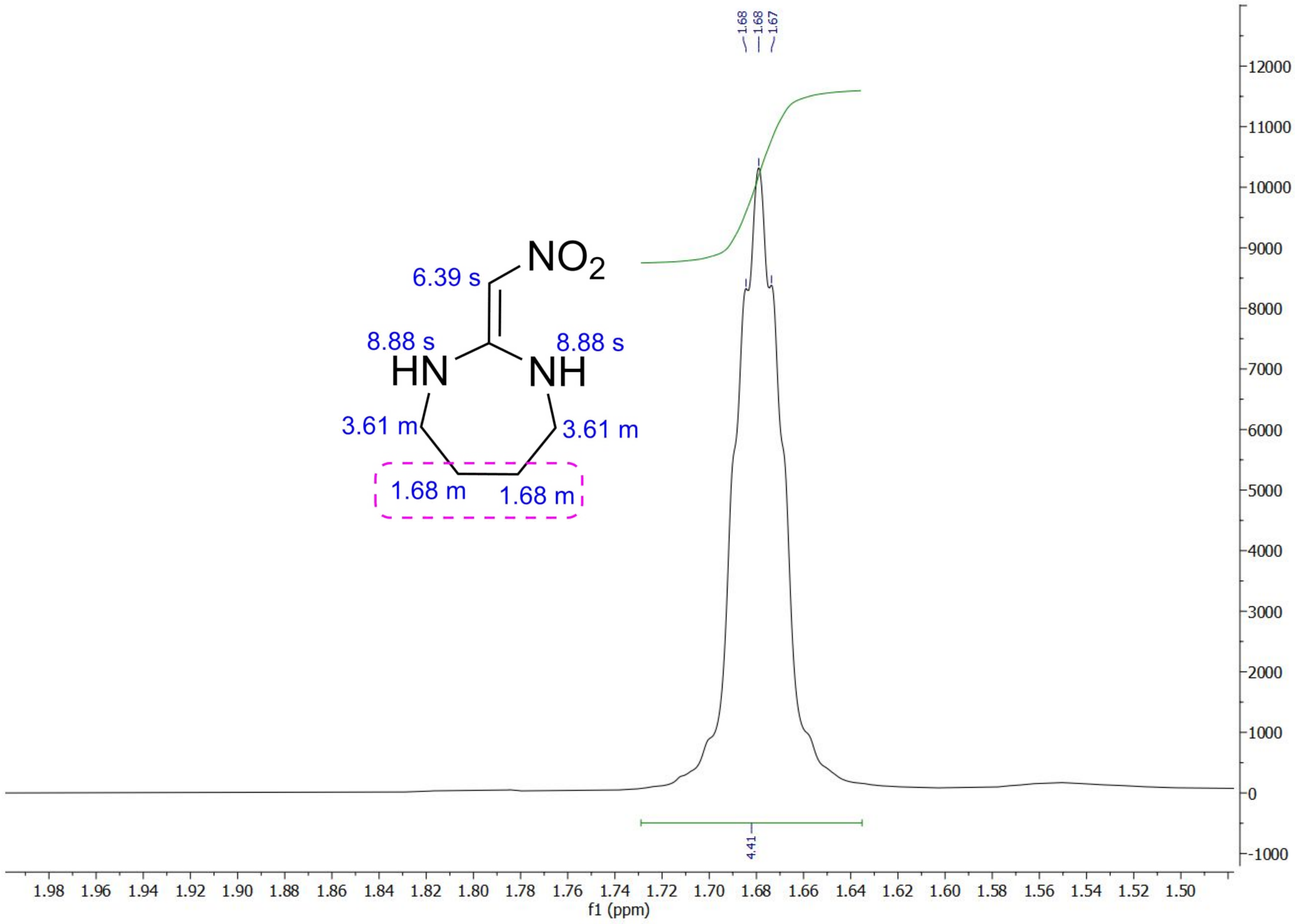

Figure S3. ^{1}H NMR (500 MHz) spectrum of 2-(nitromethylene)-1,3-diazepine in DMSO- d_6 expansion 2 (**10**).

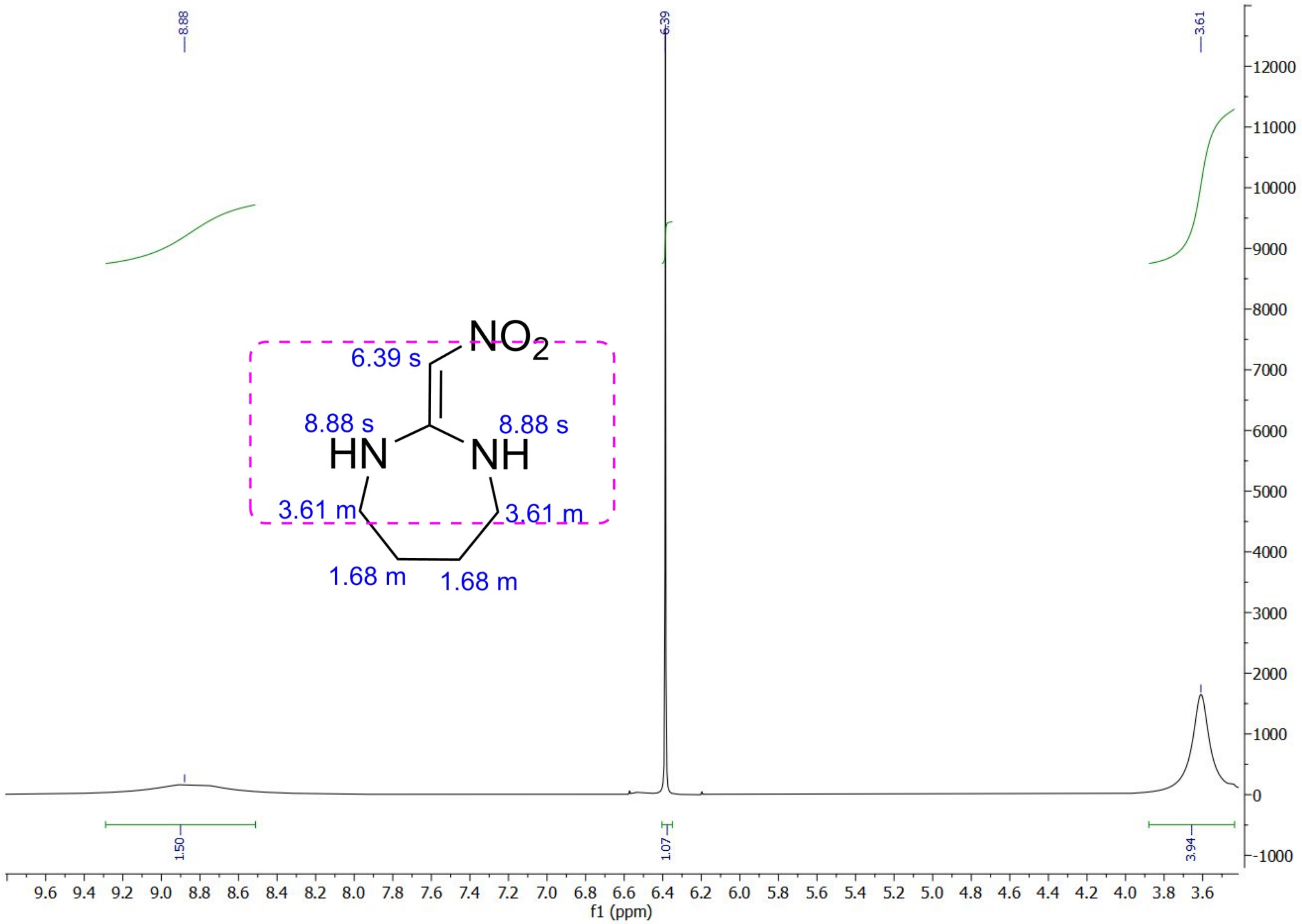

Figure S4. ^{13}C NMR (126 MHz) spectrum of 2-(nitromethylene)-1,3-diazepine in DMSO- d_6 (**10**).

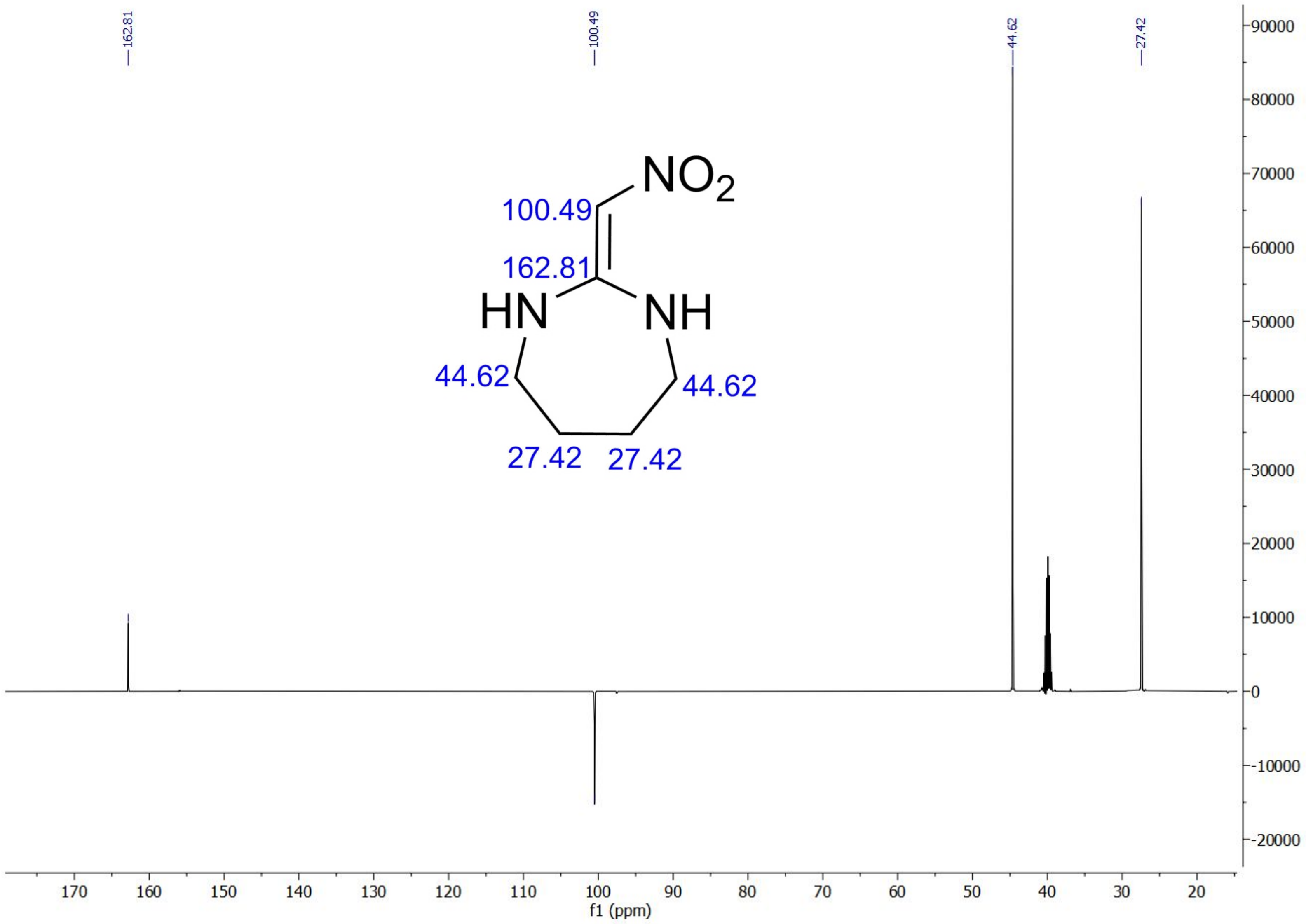

Figure S5. Mass spectrum of 2-(nitromethylene)-1,3-diazepine (**10**).

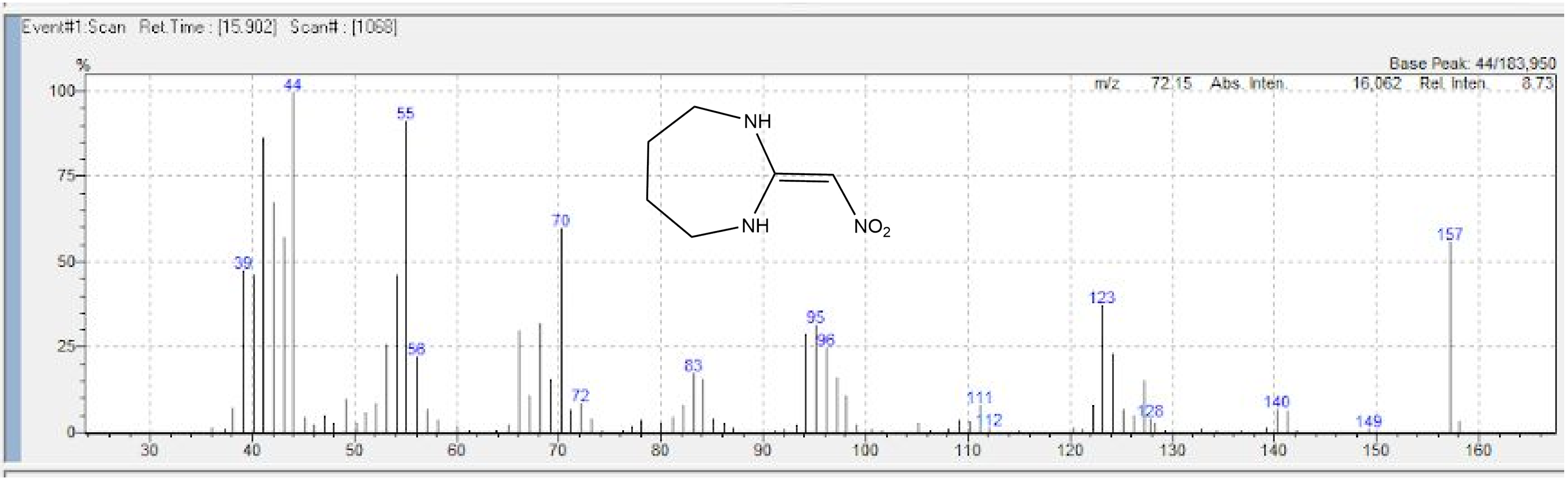

Figure S6. High-resolution mass spectrum of 2-(nitromethylene)-1,3-diazepine (**10**).

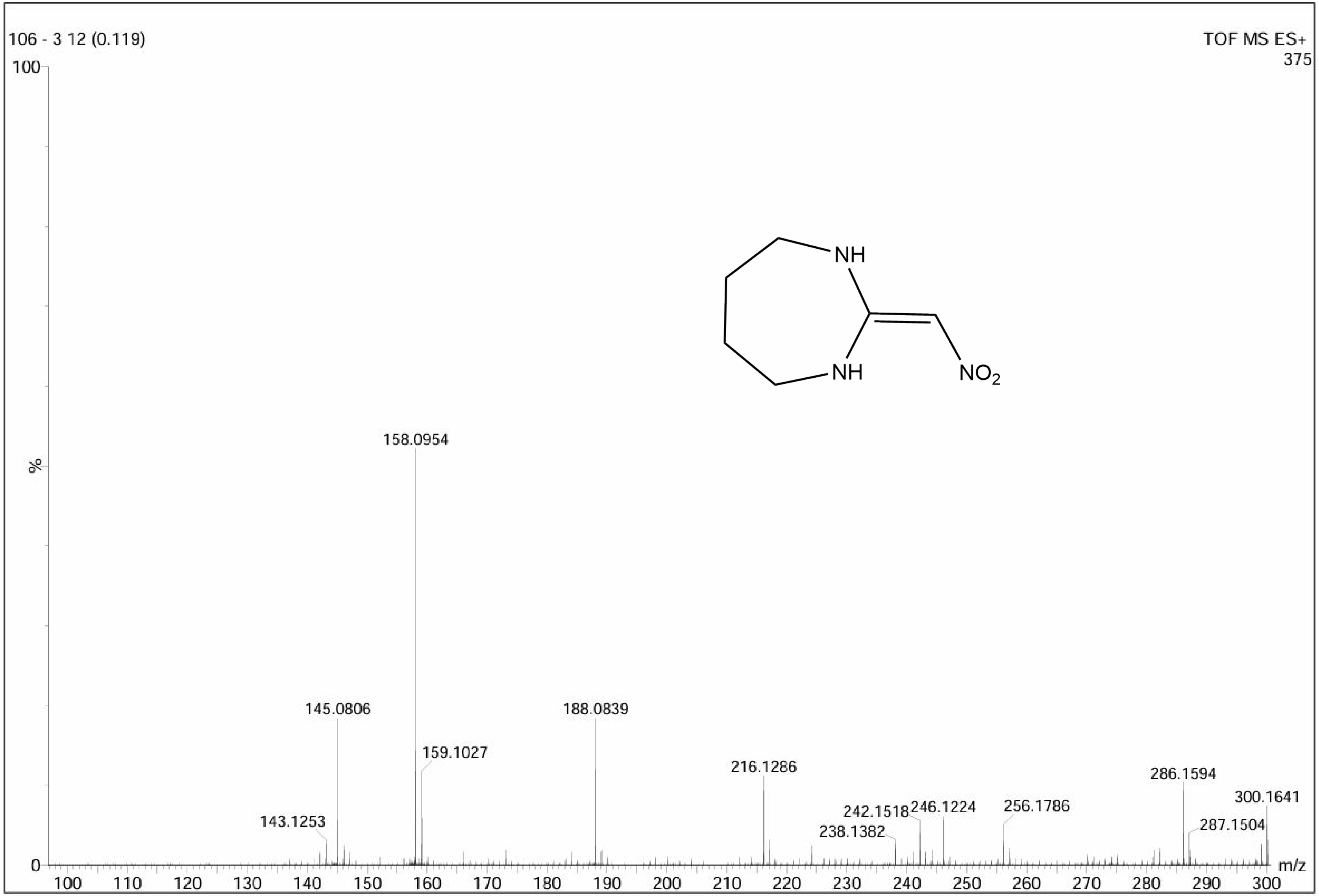

Figure S7. IR spectrum of 2-(nitromethylene)-1,3-diazepine (**10**).

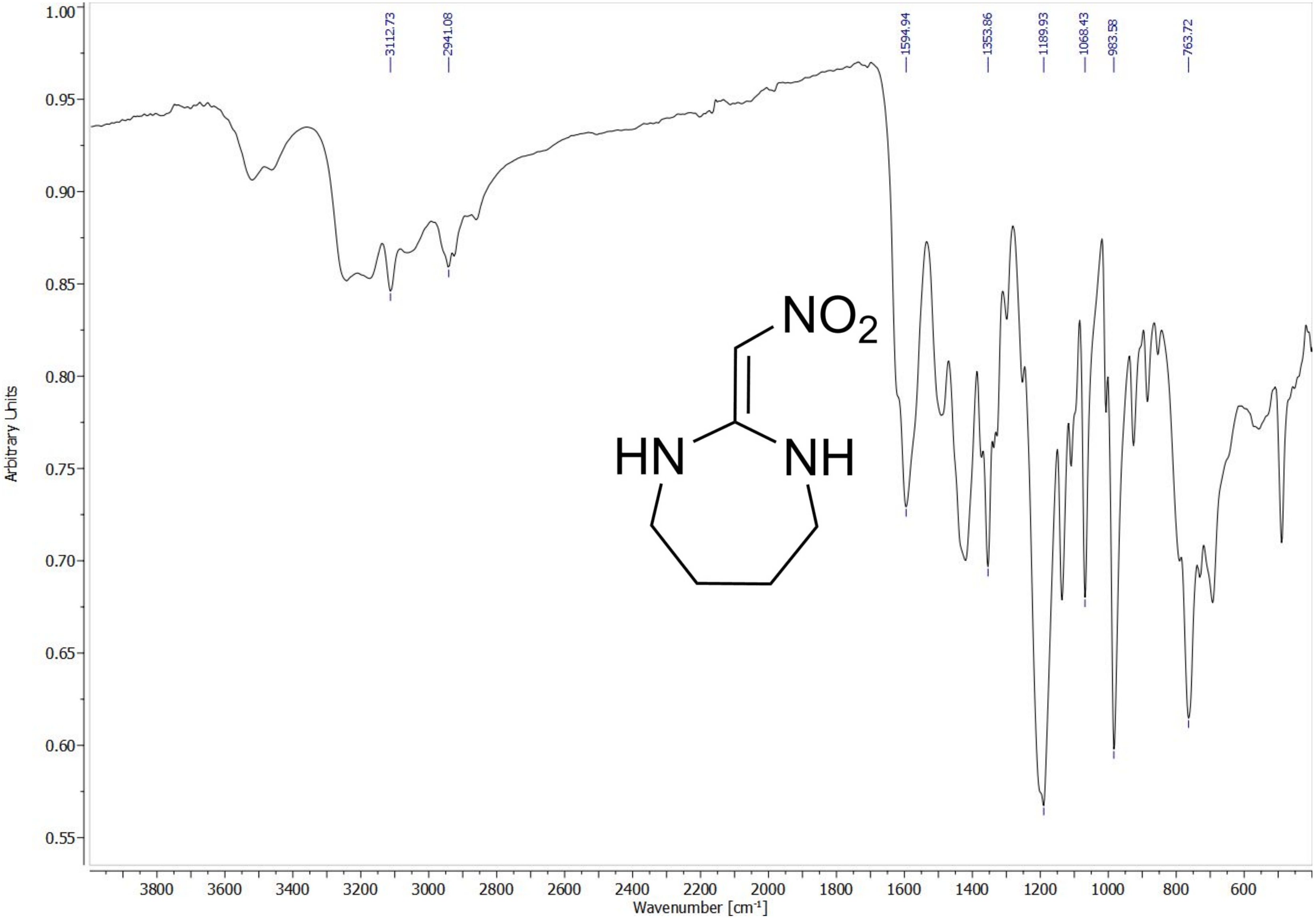

Figure S8. ^{1}H NMR (400 MHz) spectrum of 2-(1,3-diazepan-2-ylidene)malononitrile in DMSO- d_6 (**11**).

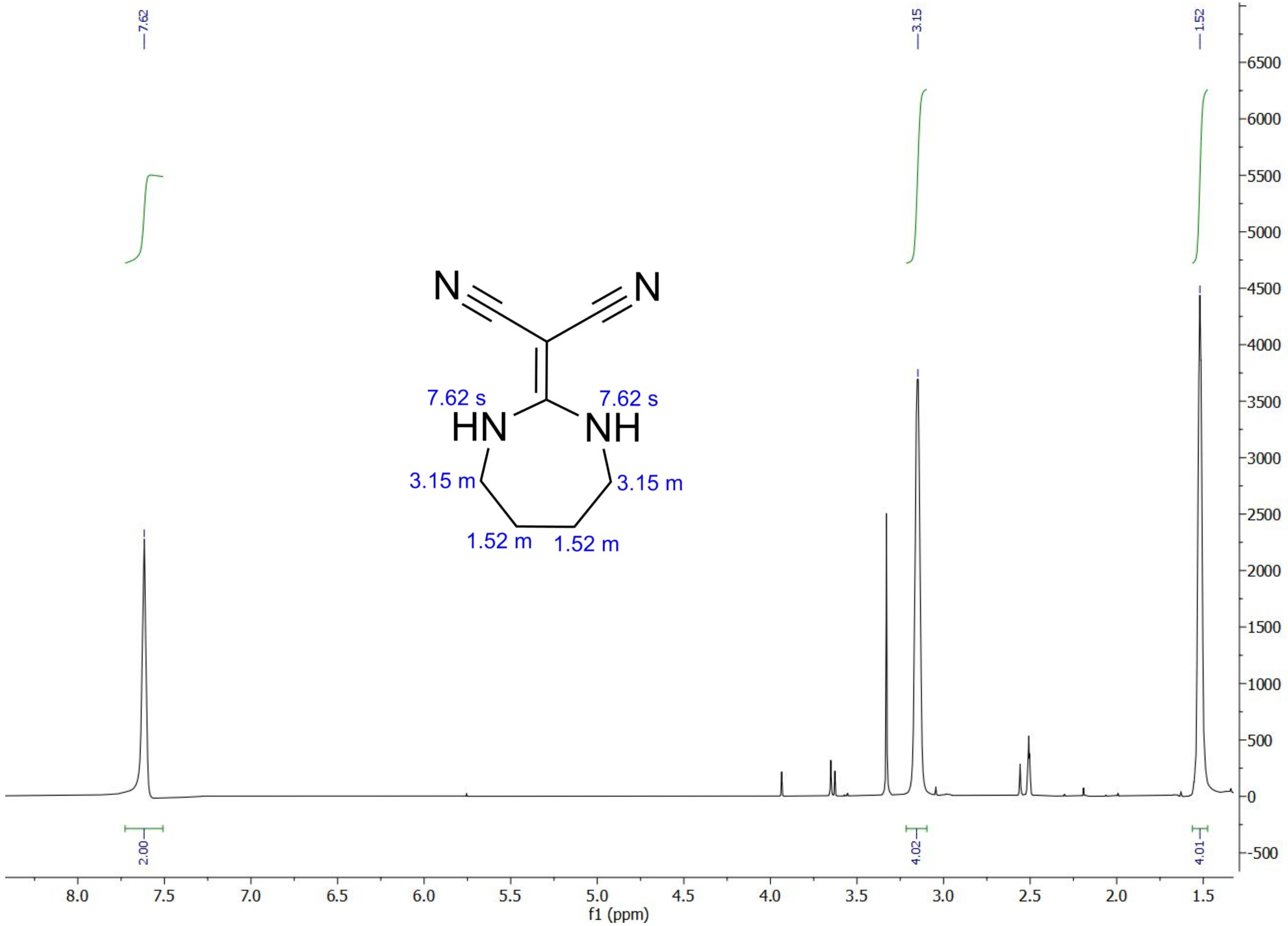

Figure S9. ^{1}H NMR (400 MHz) spectrum of 2-(1,3-diazepan-2-ylidene)malononitrile in DMSO- d_6 (expansion 1) (**11**).

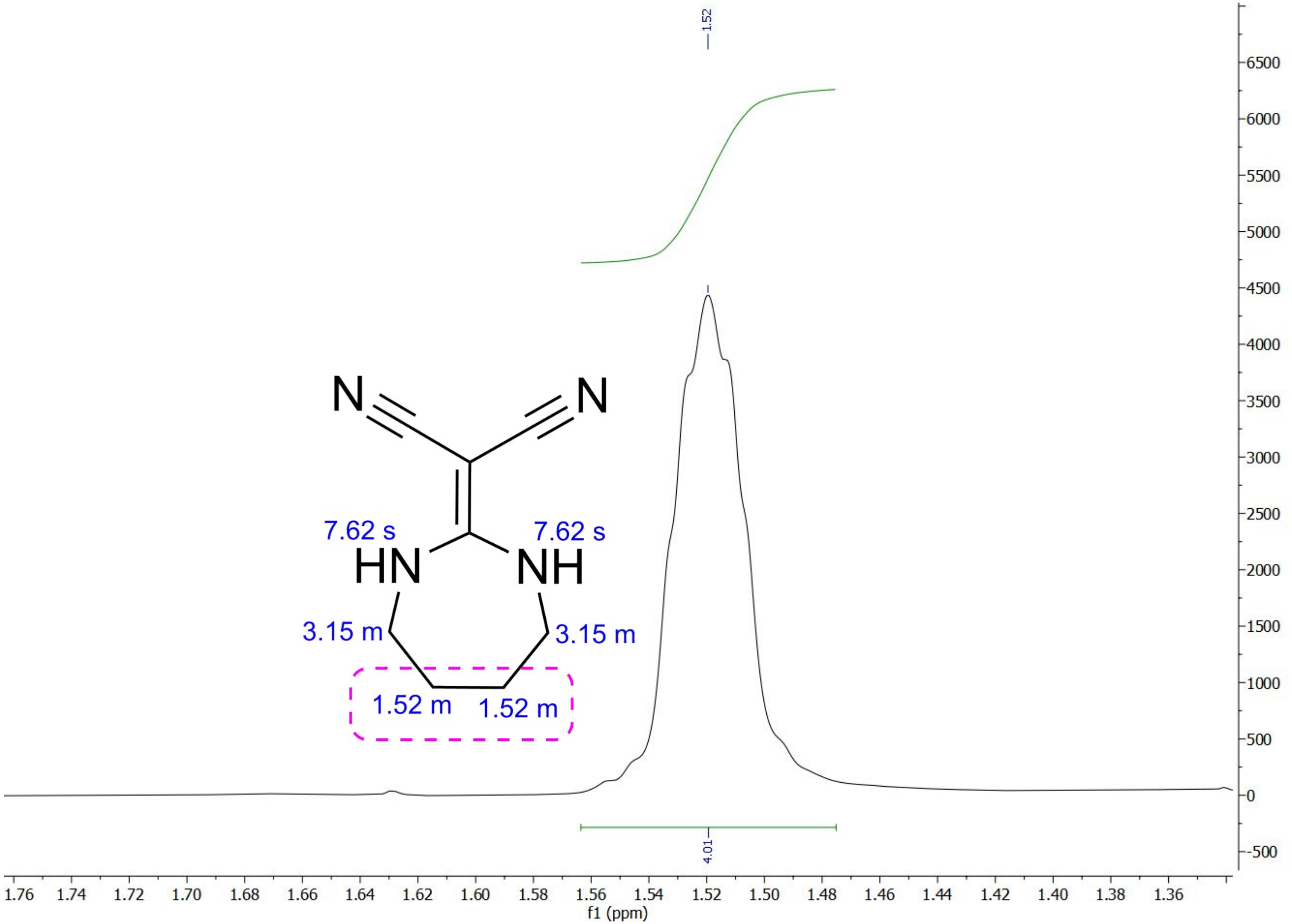

Figure S10. ^{1}H NMR (400 MHz) spectrum of 2-(1,3-diazepan-2-ylidene)malononitrile in DMSO- d_6 (expansion 2) (**11**).

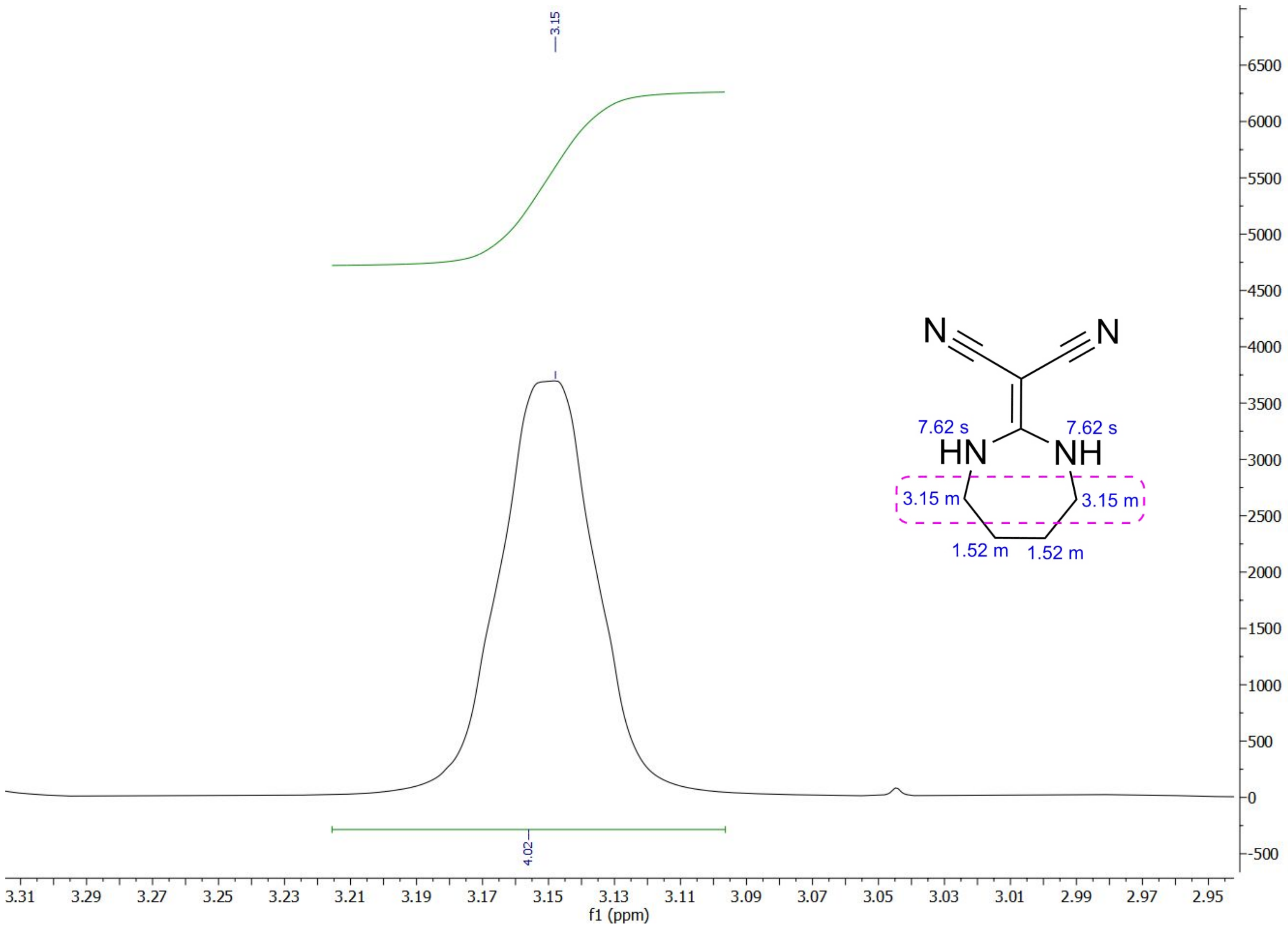

Figure S11. ^{13}C NMR (101 MHz) spectrum of 2-(1,3-diazepan-2-ylidene)malononitrile in DMSO- d_6 (**11**).

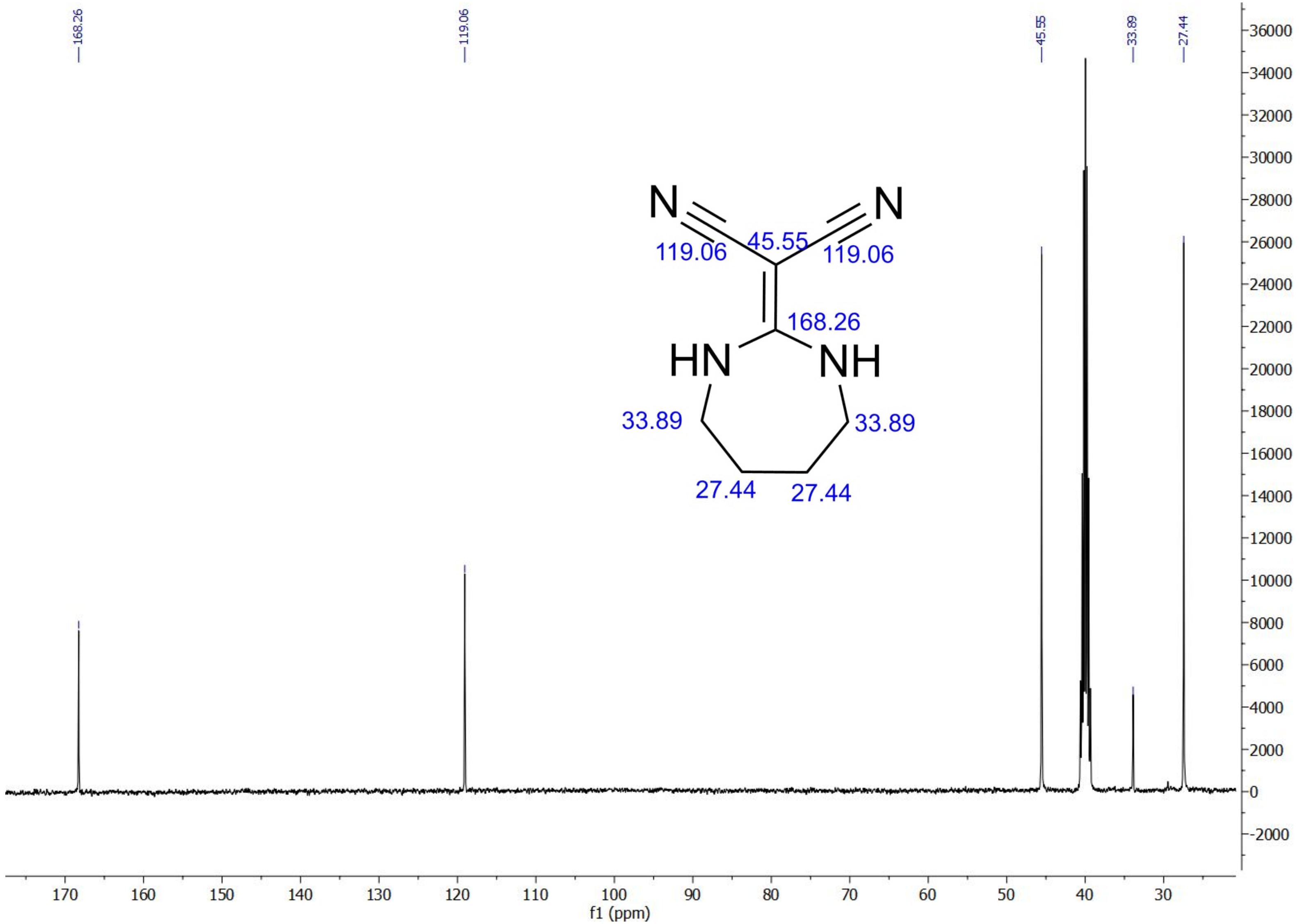

Figure S12. Mass spectrum of 2-(1,3-diazepan-2-ylidene)malononitrile (**11**).

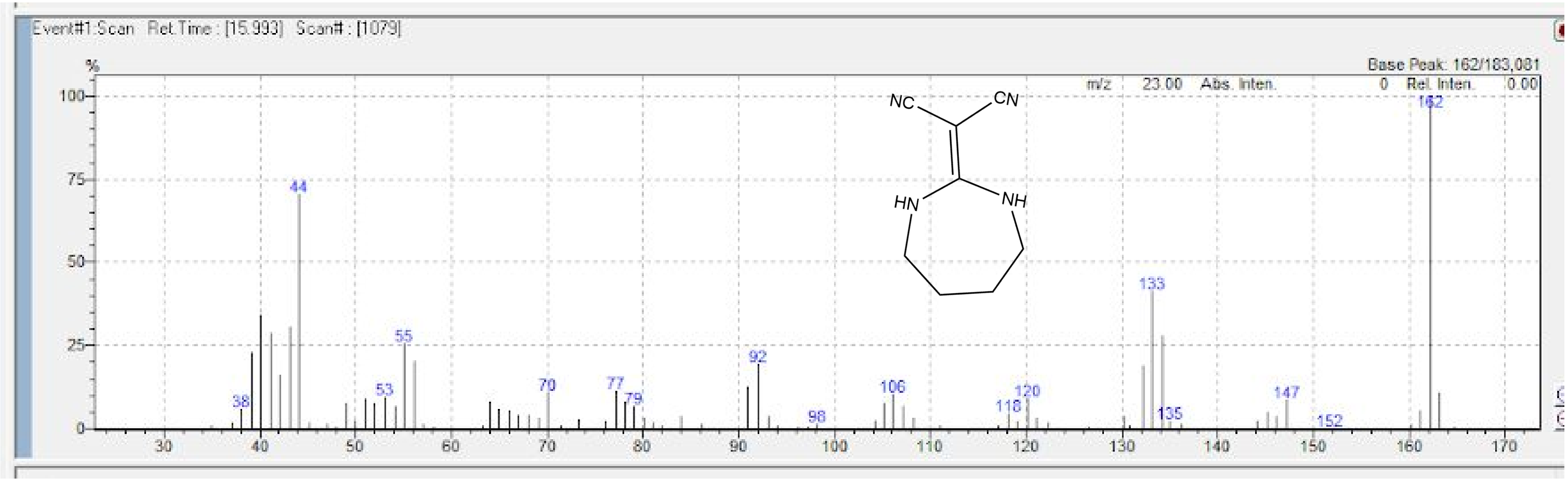

Figure S13. High-resolution mass spectrum of 2-(1,3-diazepan-2-ylidene)malononitrile (**11**).

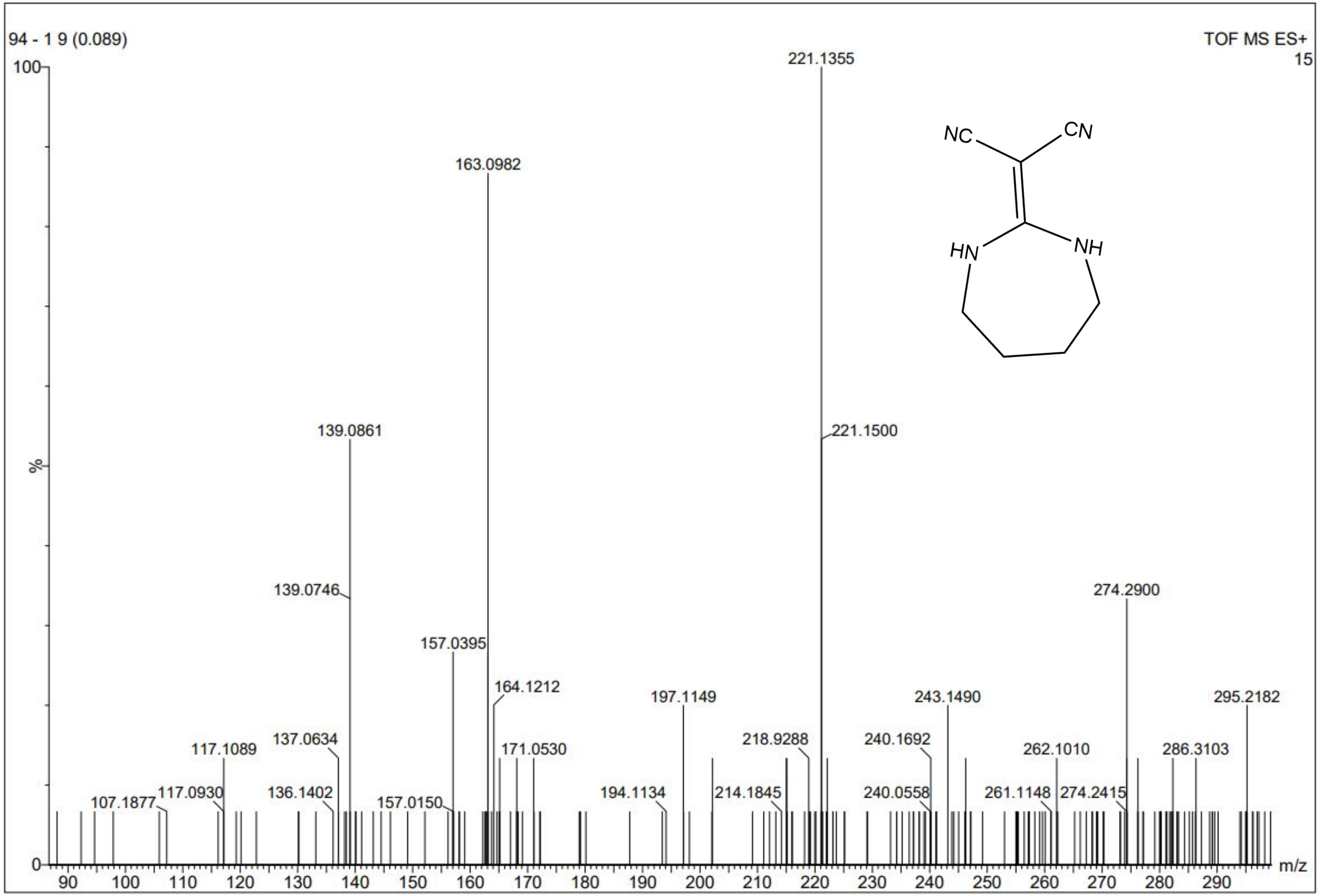

Figure S14. IR spectrum of 2-(1,3-diazepan-2-ylidene)malononitrile (**11**).

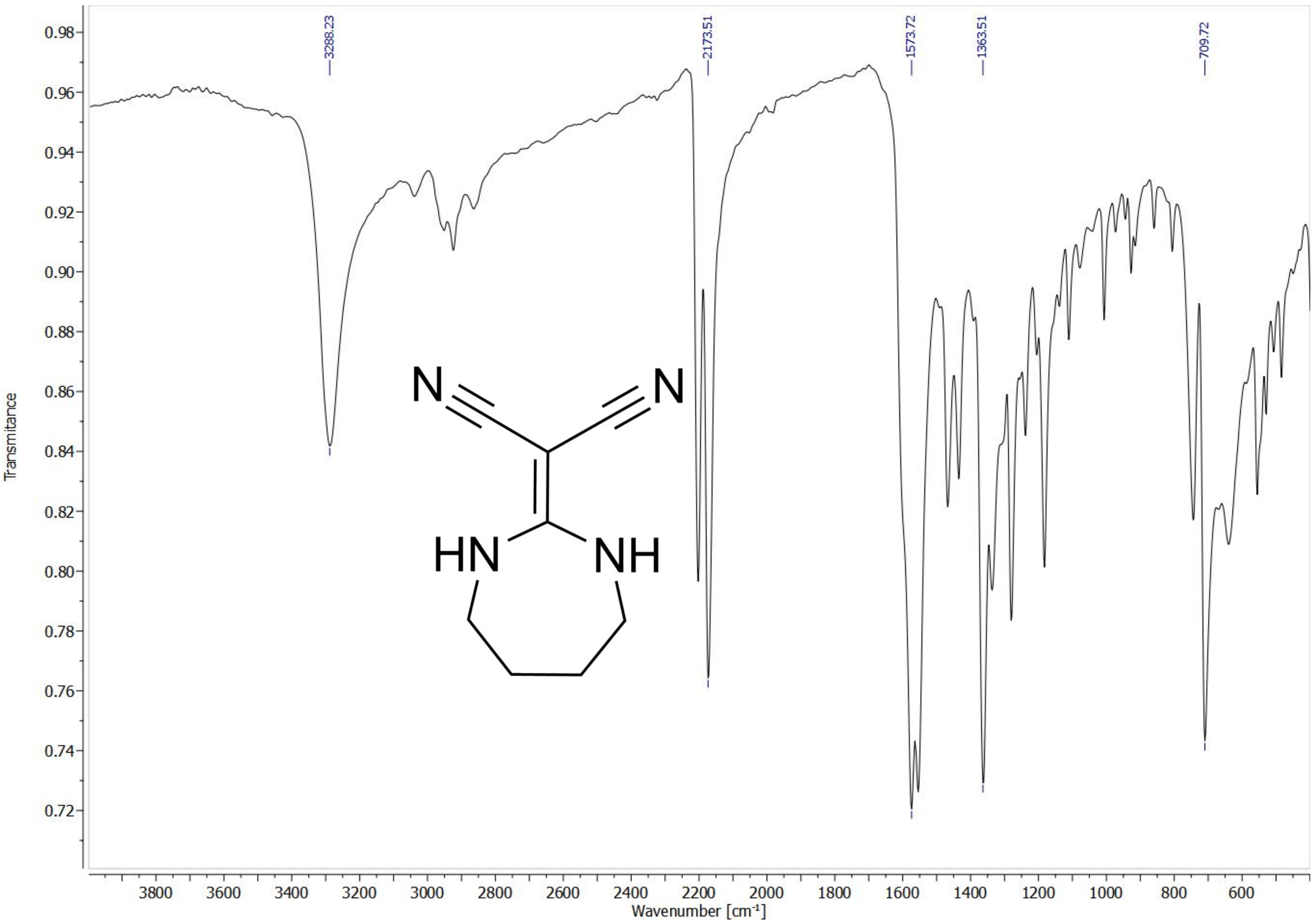

Figure S15. ^{1}H NMR (400 MHz) spectrum of methyl 2-cyano-2-(1,3-diazepan-2-ylidene)acetate in DMSO- d_6 (**12**).

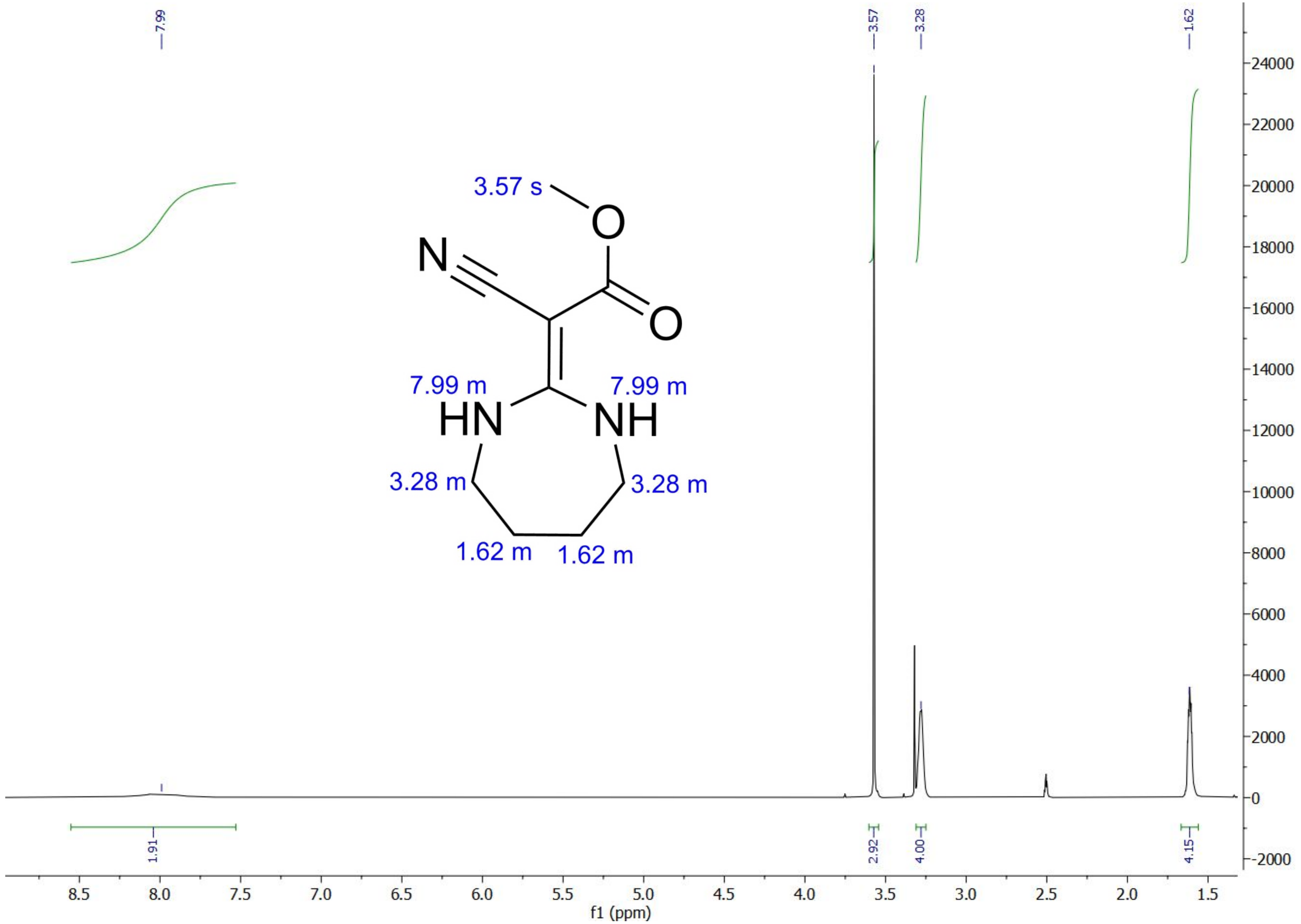

Figure S16. ^{1}H NMR (400 MHz) spectrum of methyl 2-cyano-2-(1,3-diazepan-2-ylidene)acetate in DMSO- d_6 expansion 1 (**12**).

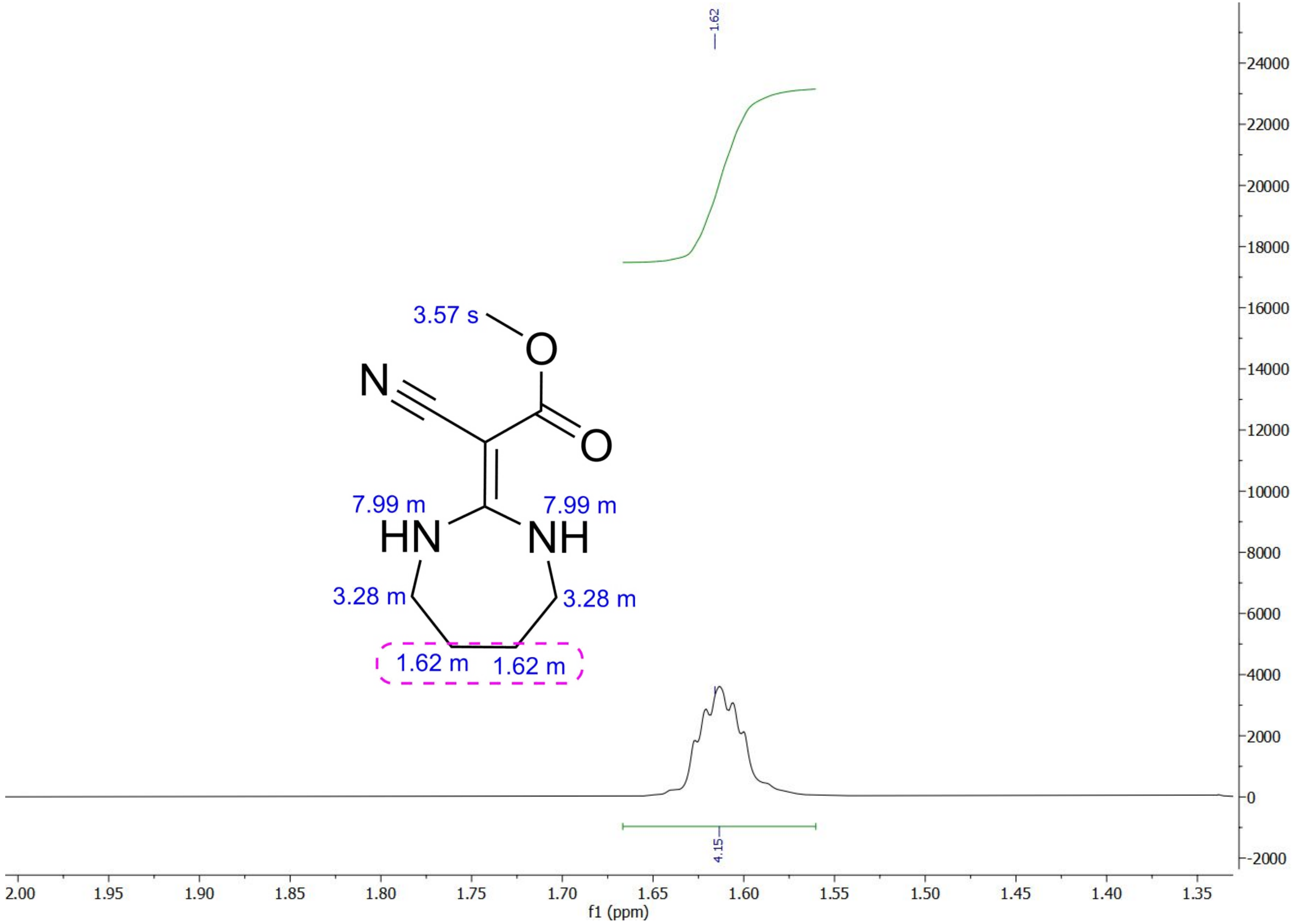

Figure S17. ^{1}H NMR (400 MHz) spectrum of methyl 2-cyano-2-(1,3-diazepan-2-ylidene)acetate in DMSO- d_6 expansion 2 (**12**).

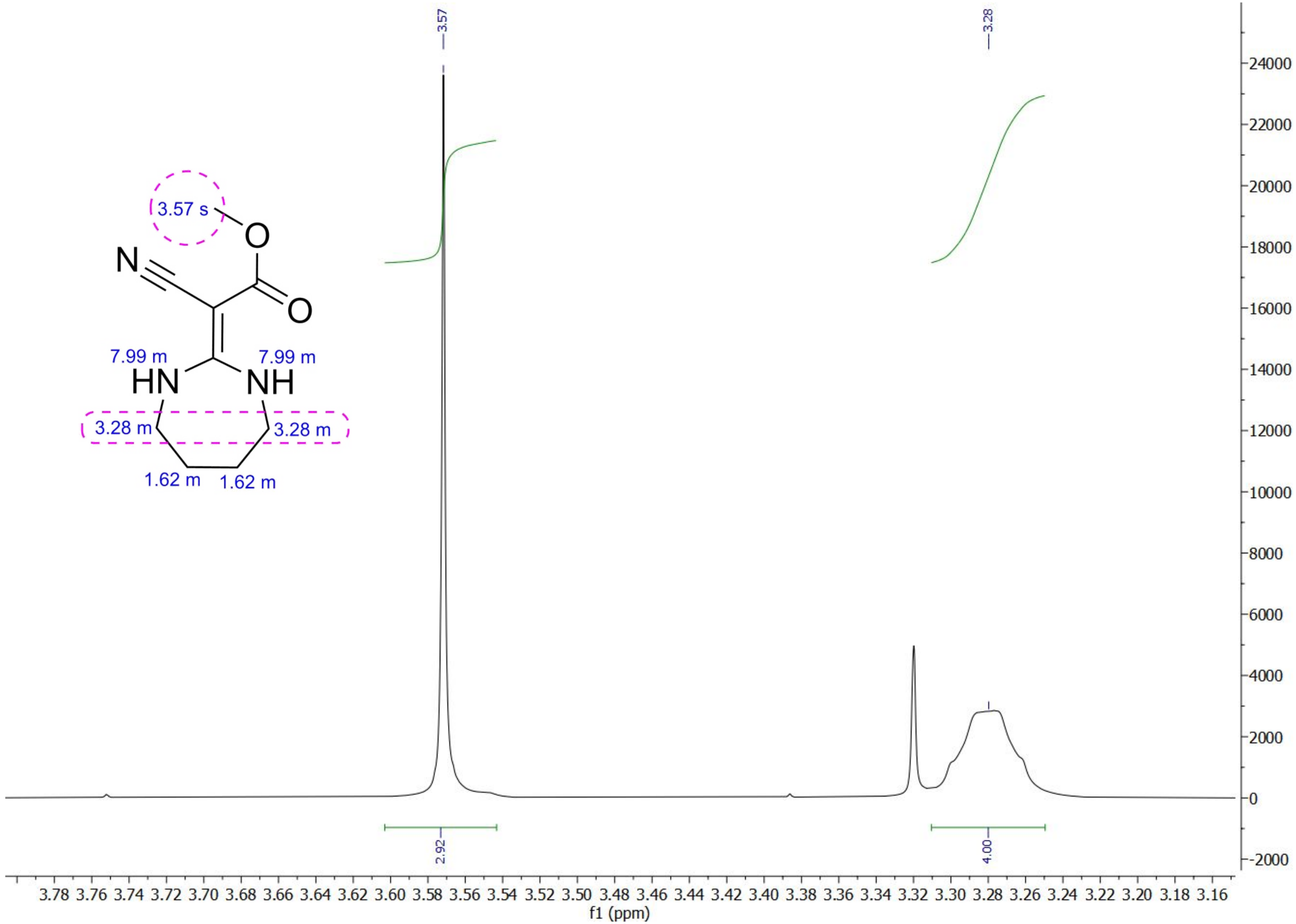

Figure S18. ^{13}C NMR (101 MHz) spectrum of methyl 2-cyano-2-(1,3-diazepan-2-ylidene)acetate in DMSO- d_6 (**12**).

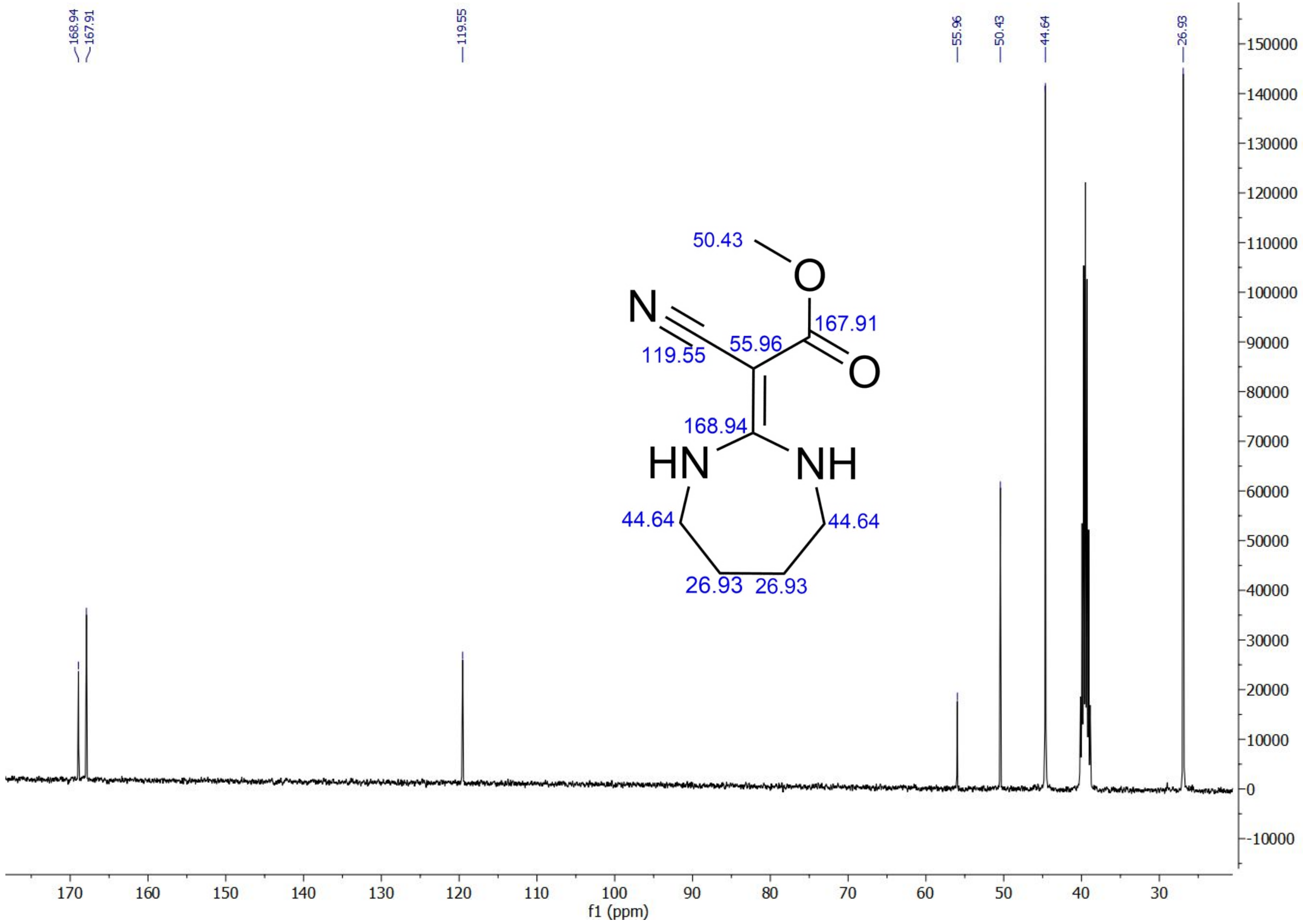

Figure S19. Mass spectrum of methyl 2-cyano-2-(1,3-diazepan-2-ylidene)acetate (**12**).

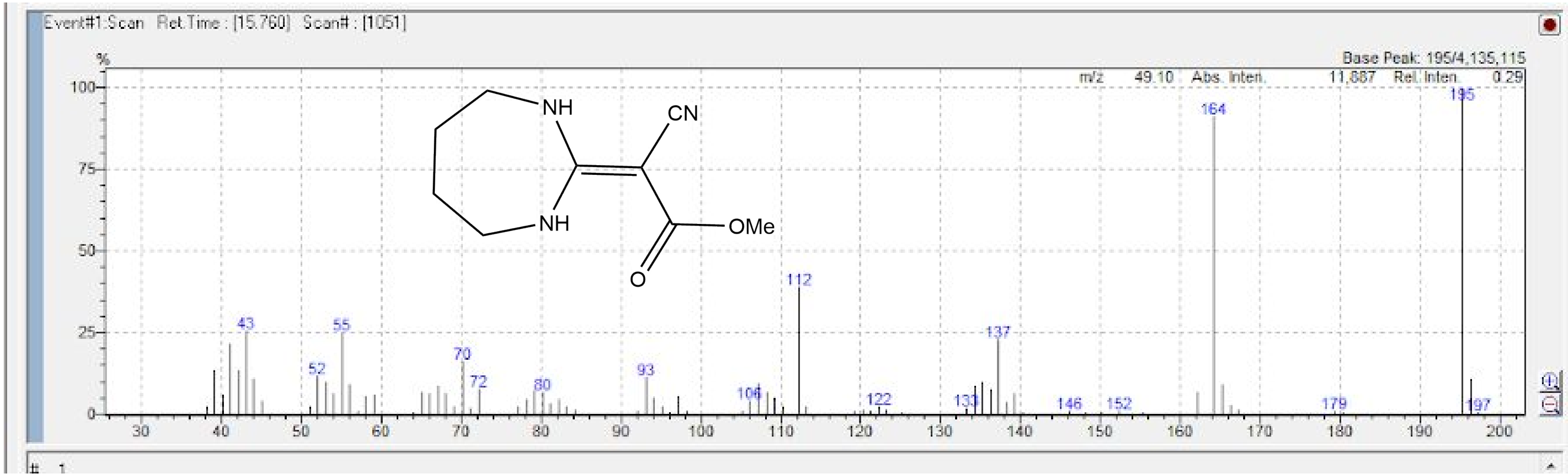

Figure S20. High-resoluton mass spectrum of methyl 2-cyano-2-(1,3-diazepan-2-ylidele)acetate (**12**).

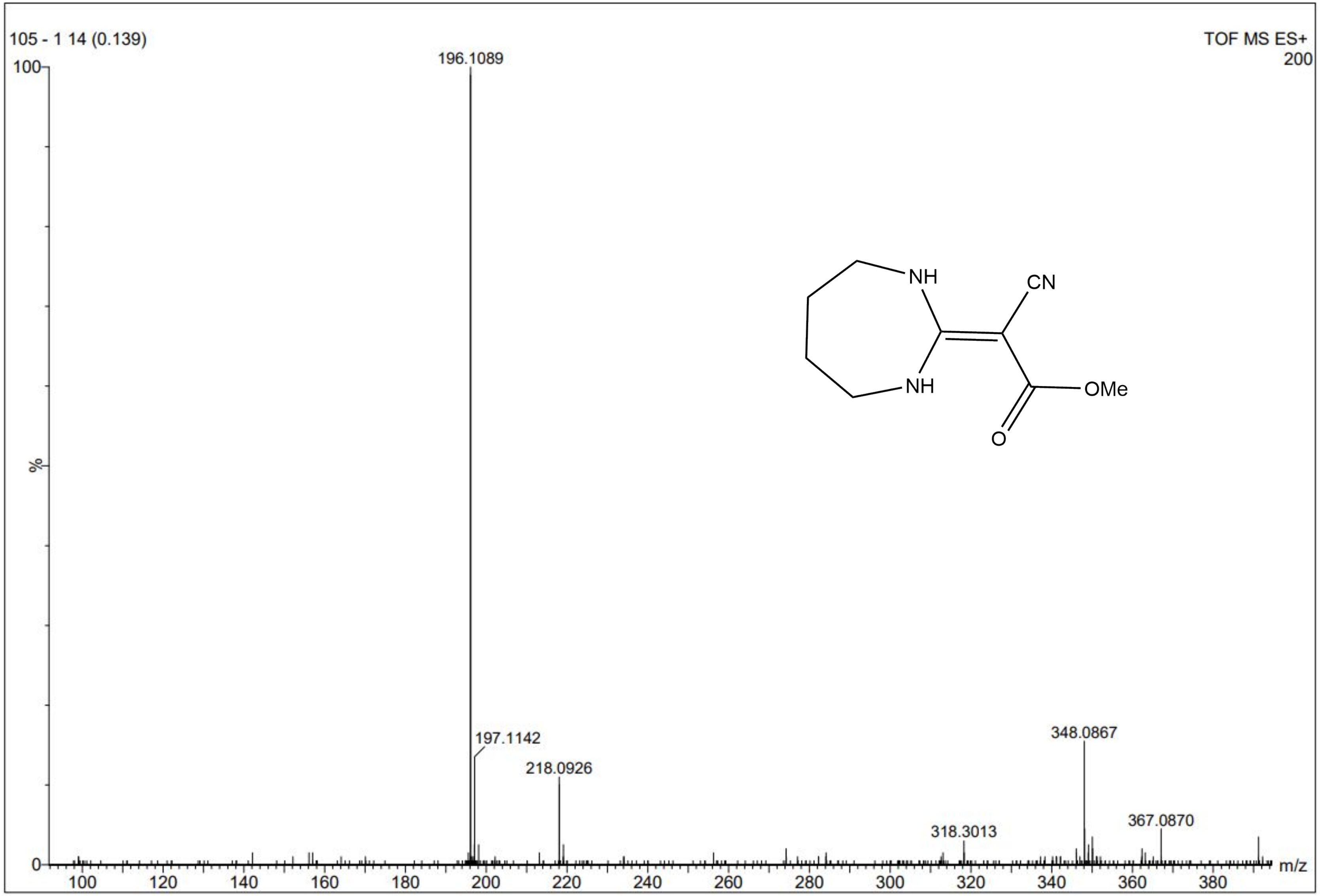

Figure S21. IR spectrum of methyl 2-cyano-2-(1,3-diazepan-2-ylidene)acetate (**12**).

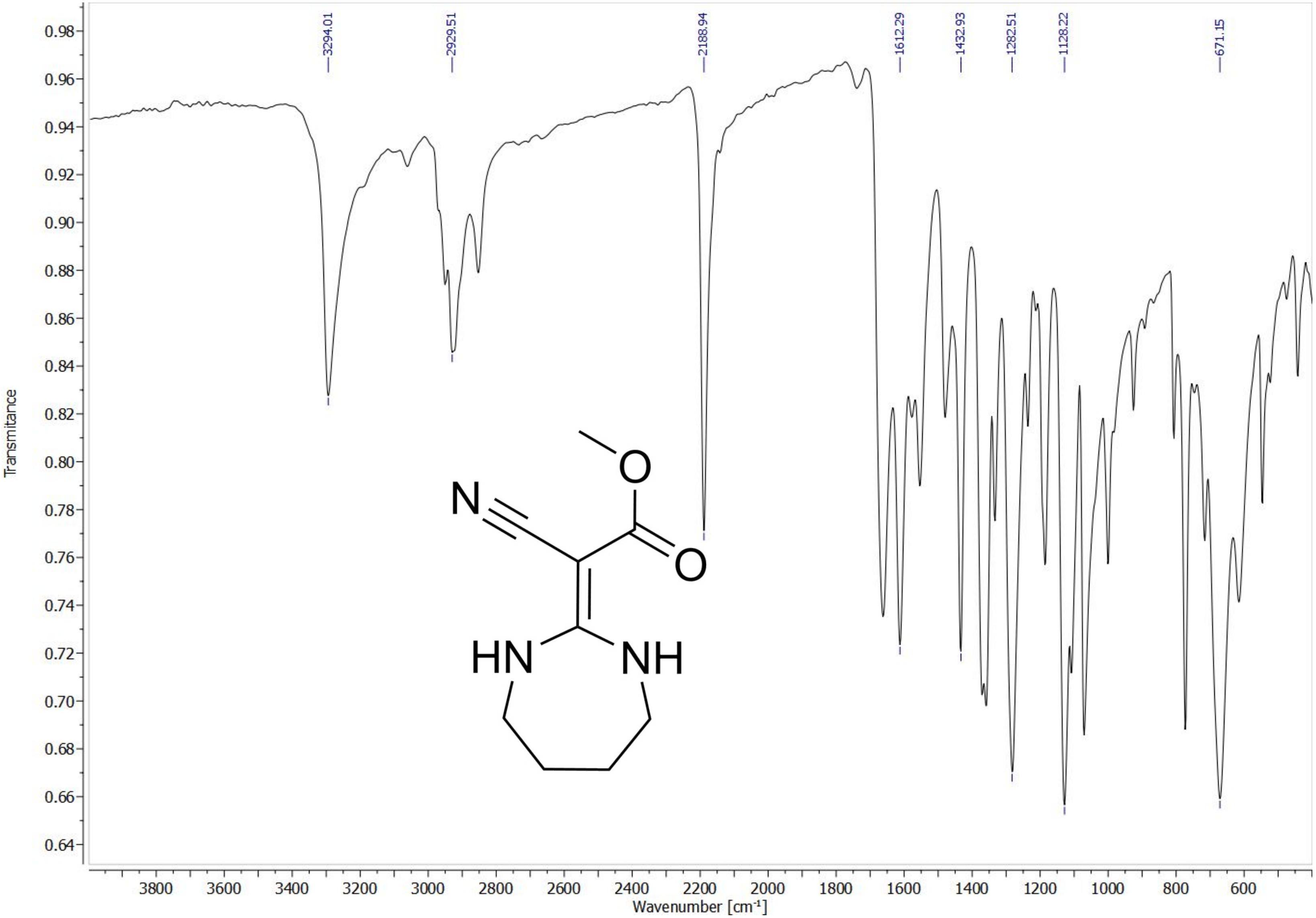

Figure S22. ^{1}H NMR (400 MHz) spectrum of diethyl 2-(1,3-diazepan-2-ylidene)malonate in DMSO- d_6 (**13**).

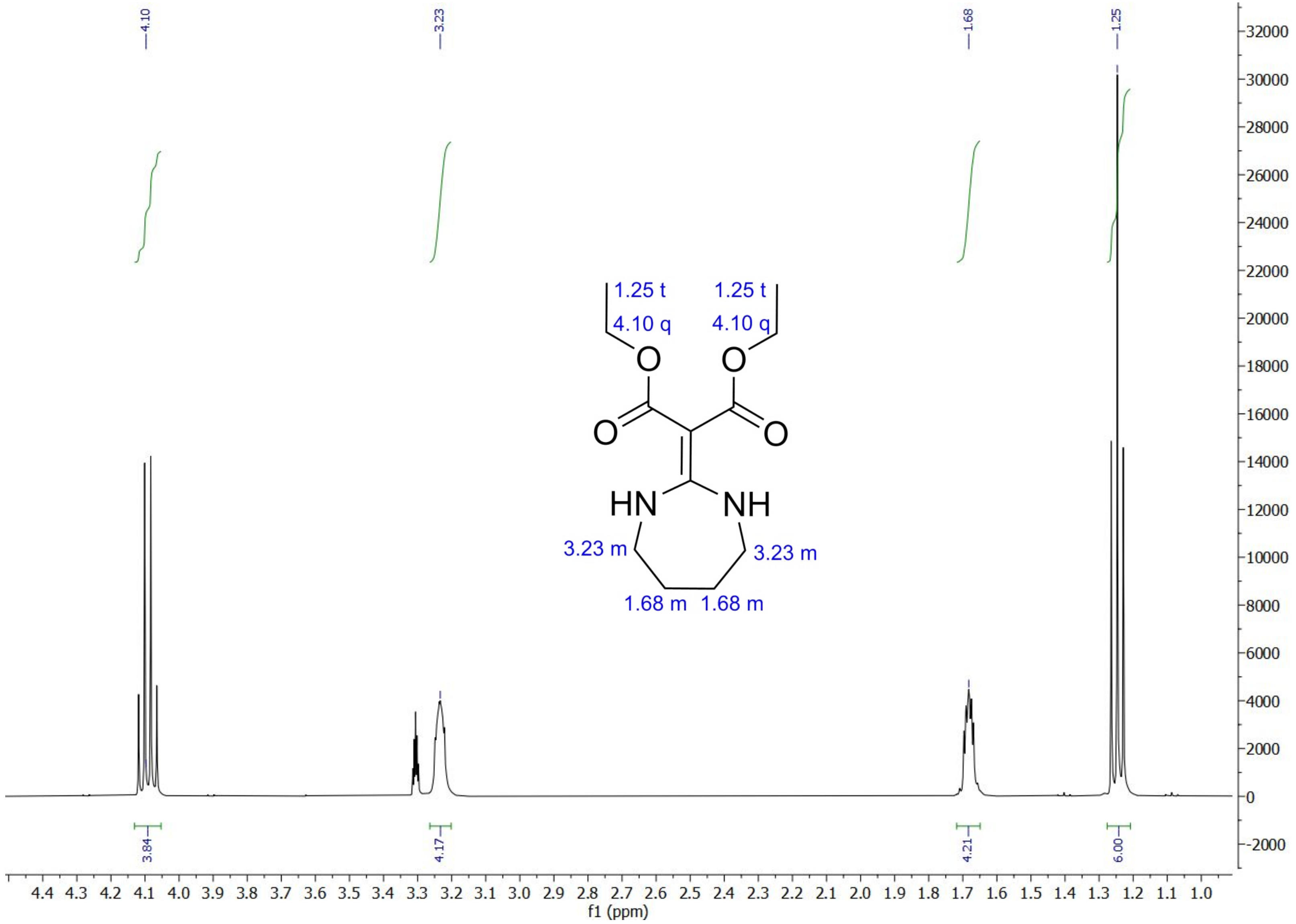

Figure S23. ^{13}C NMR (101 MHz) spectrum of diethyl 2-(1,3-diazepan-2-ylidene)malonate in DMSO- d_6 (**13**).

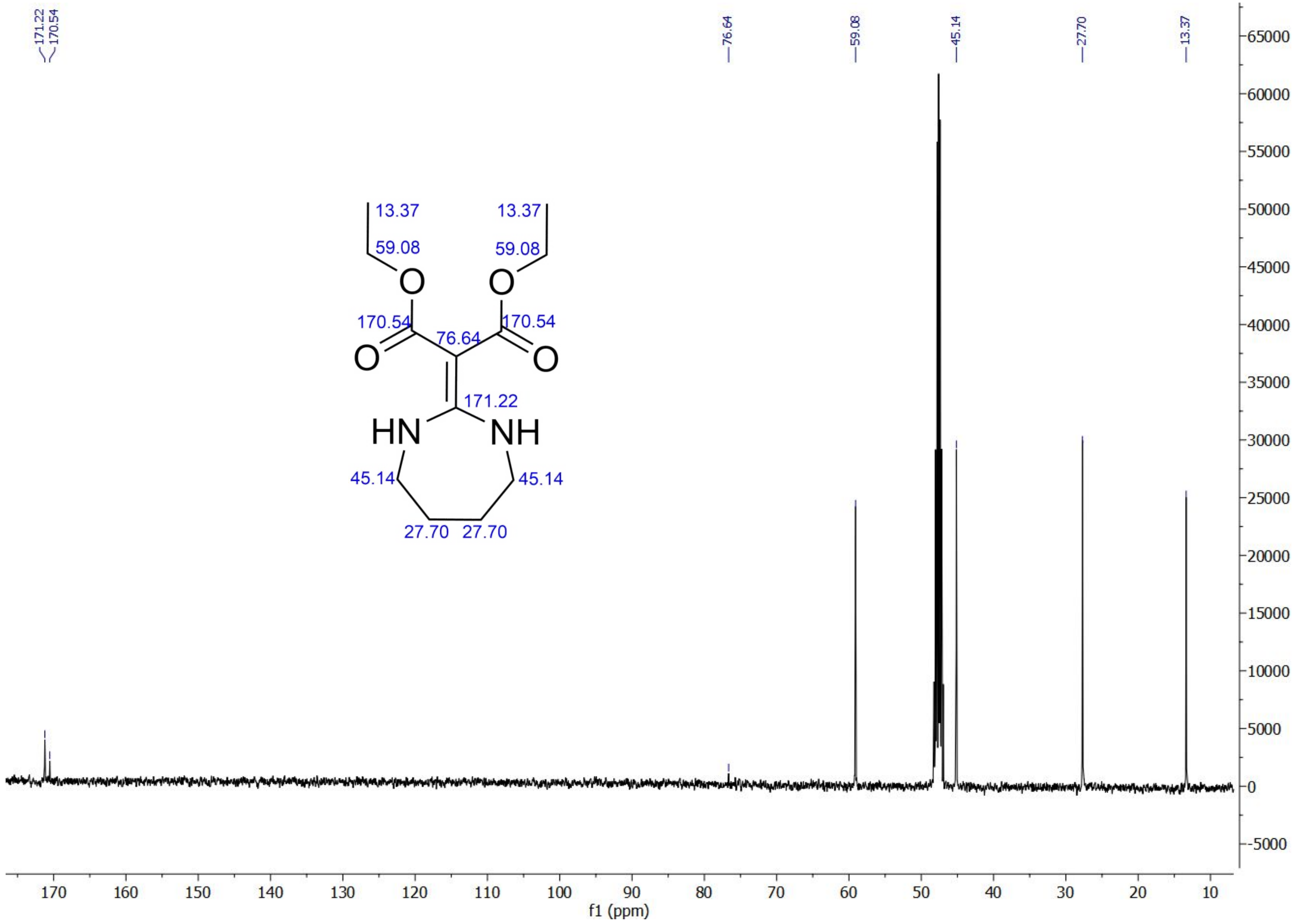

Figure S24. High-resolution mass spectrum of diethyl 2-(1,3-diazepan-2-ylidene)malonate (**13**).

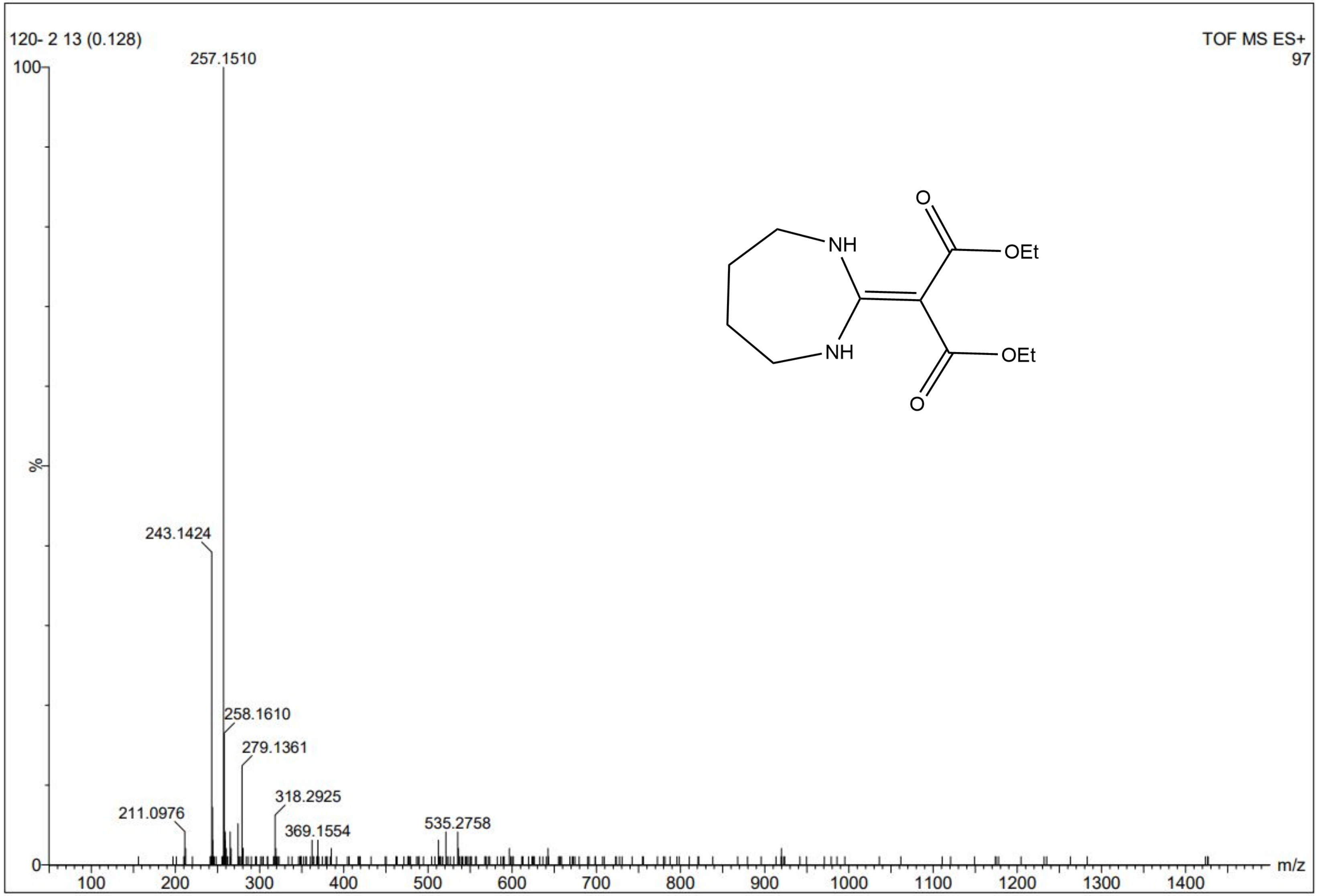

Figure S25. IR spectrum of diethyl 2-(1,3-diazepan-2-ylidene)malonate (**13**).

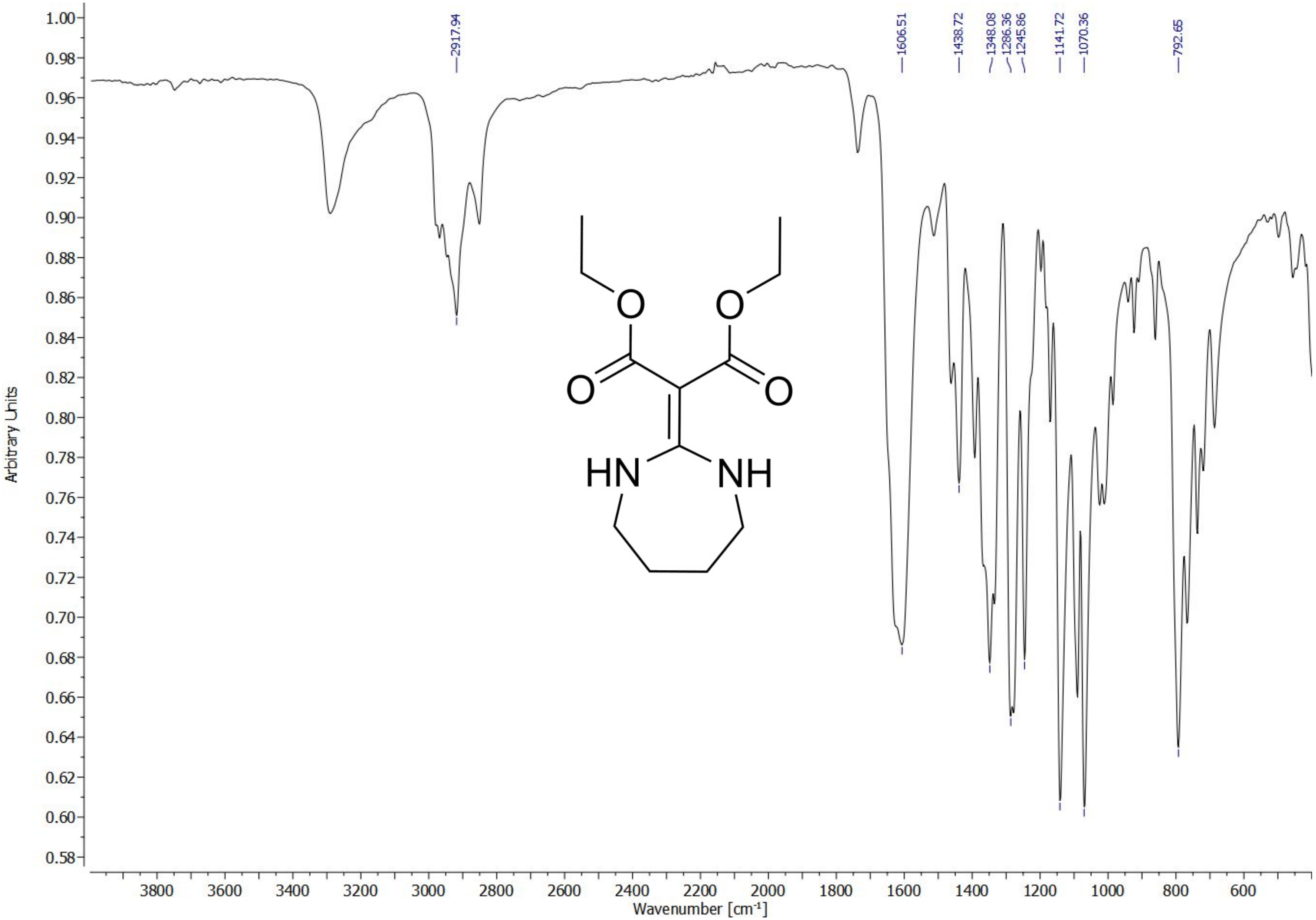

Figure S26. ^{1}H NMR (400 MHz) spectrum of 2-(1,3-diazepan-2-ylidene)-3-oxo-3-phenylpropanenitrile in $CDCl_3$ (**14**).

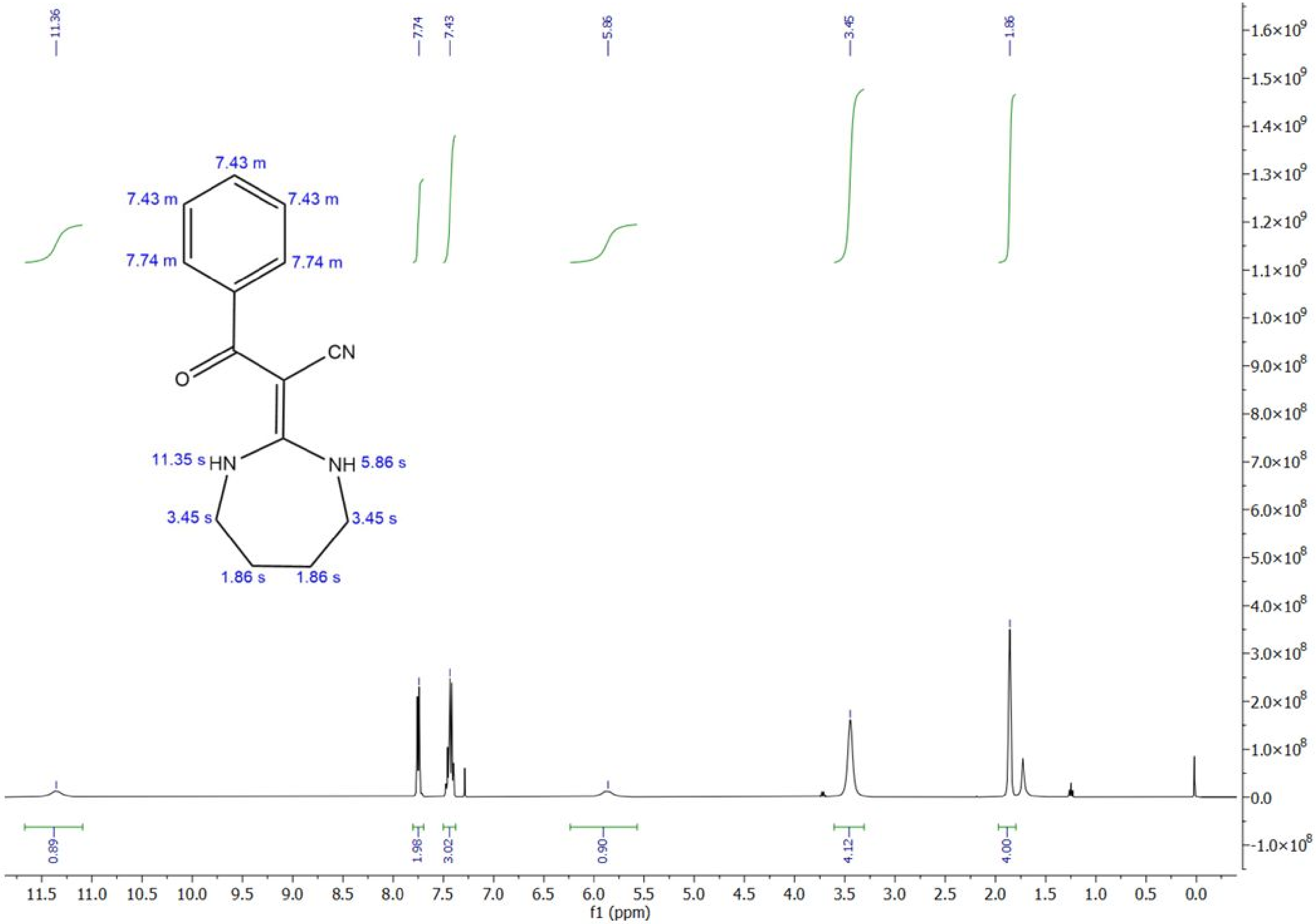

Figure S27. ^{1}H NMR (400 MHz) spectrum of 2-(1,3-diazepan-2-ylidene)-3-oxo-3-phenylpropanenitrile in $CDCl_3$ expansion 1 (**14**).

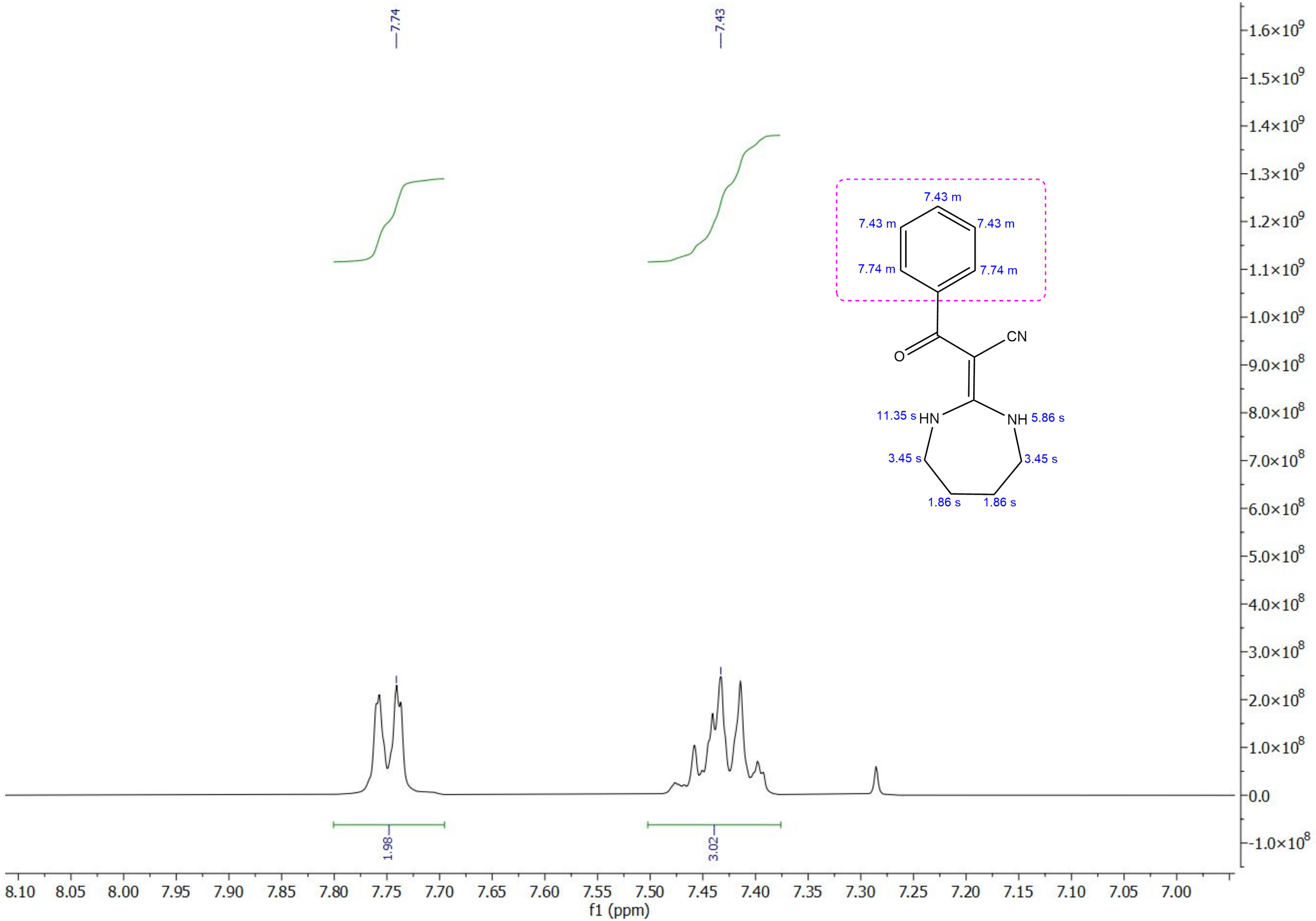

Figure S28. ^{13}C NMR (101 MHz) spectrum of 2-(1,3-diazepan-2-ylidene)-3-oxo-3-phenylpropanenitrile in $CDCl_3$ (**14**).

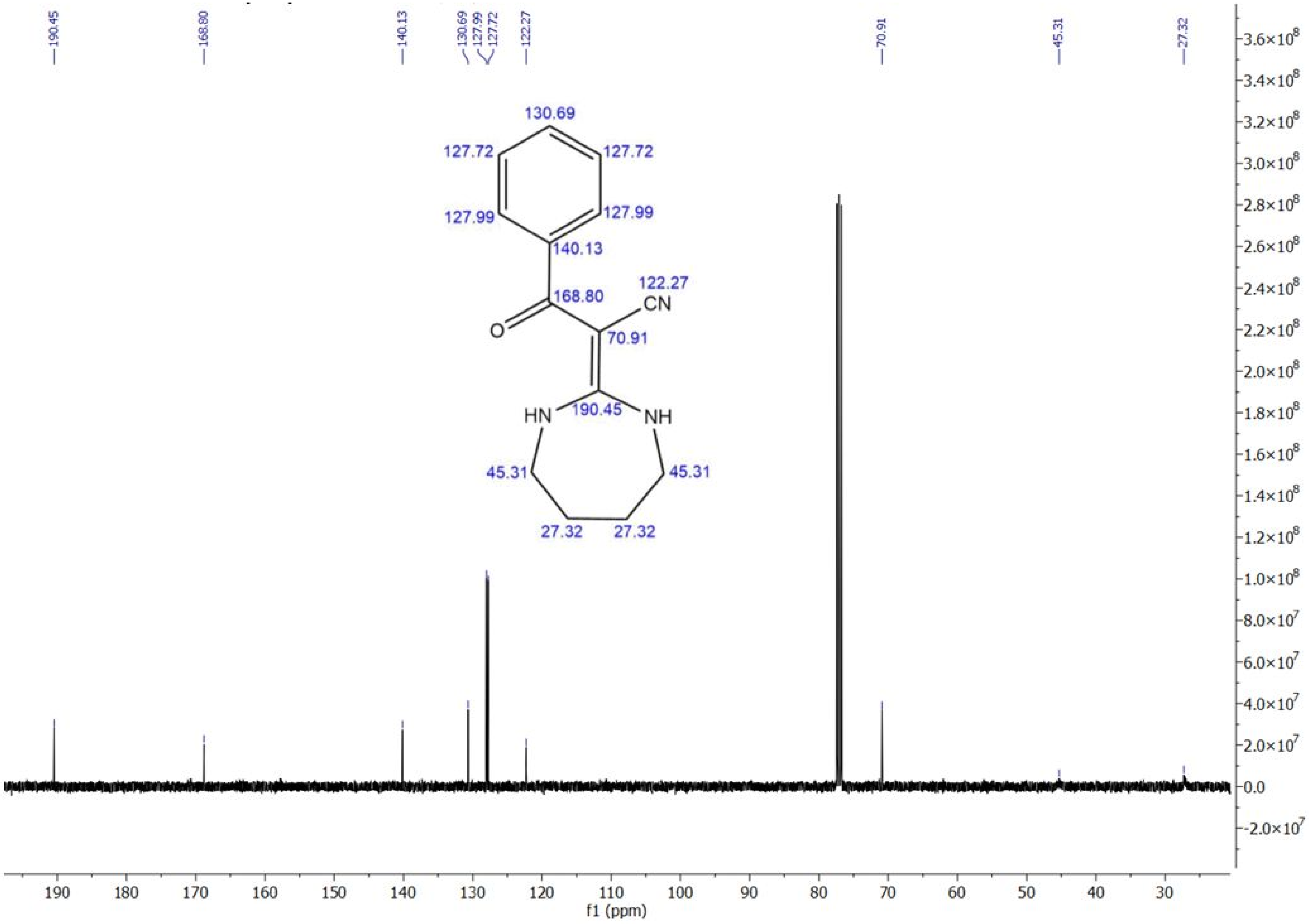

Figure S29. Mass spectrum of 2-(1,3-diazepan-2-ylidene)-3-oxo-3-phenylpropanenitrile (**14**).

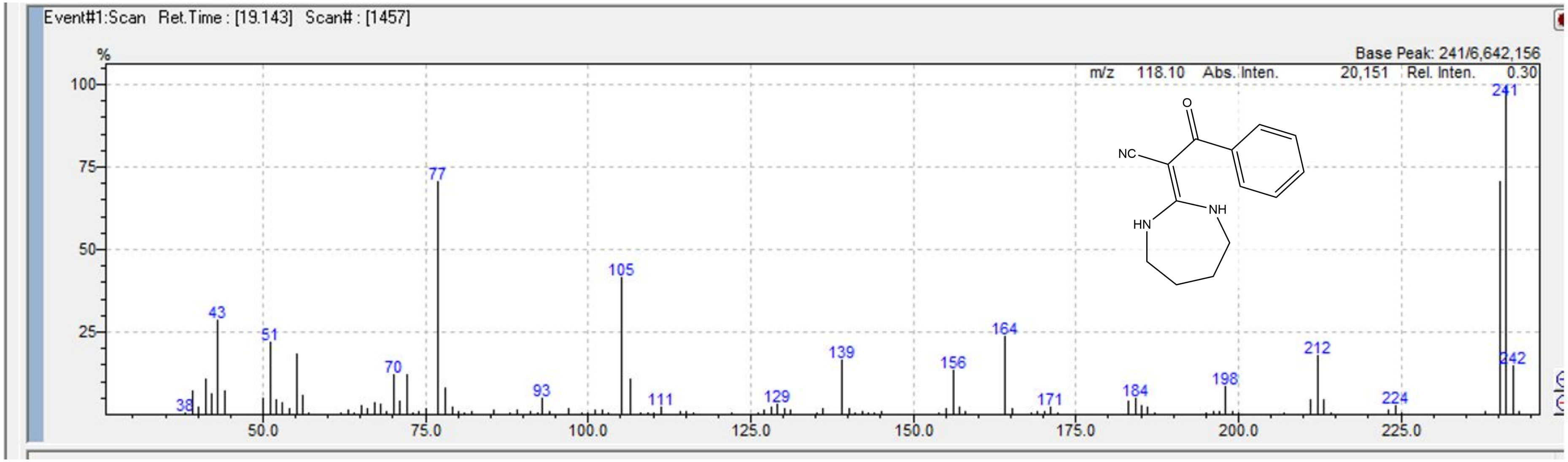

Figure S30. High-resolution mass spectrum of 2-(1,3-diazepan-2-ylidele)-3-oxo-3-phenylpropanenitrile (**14**).

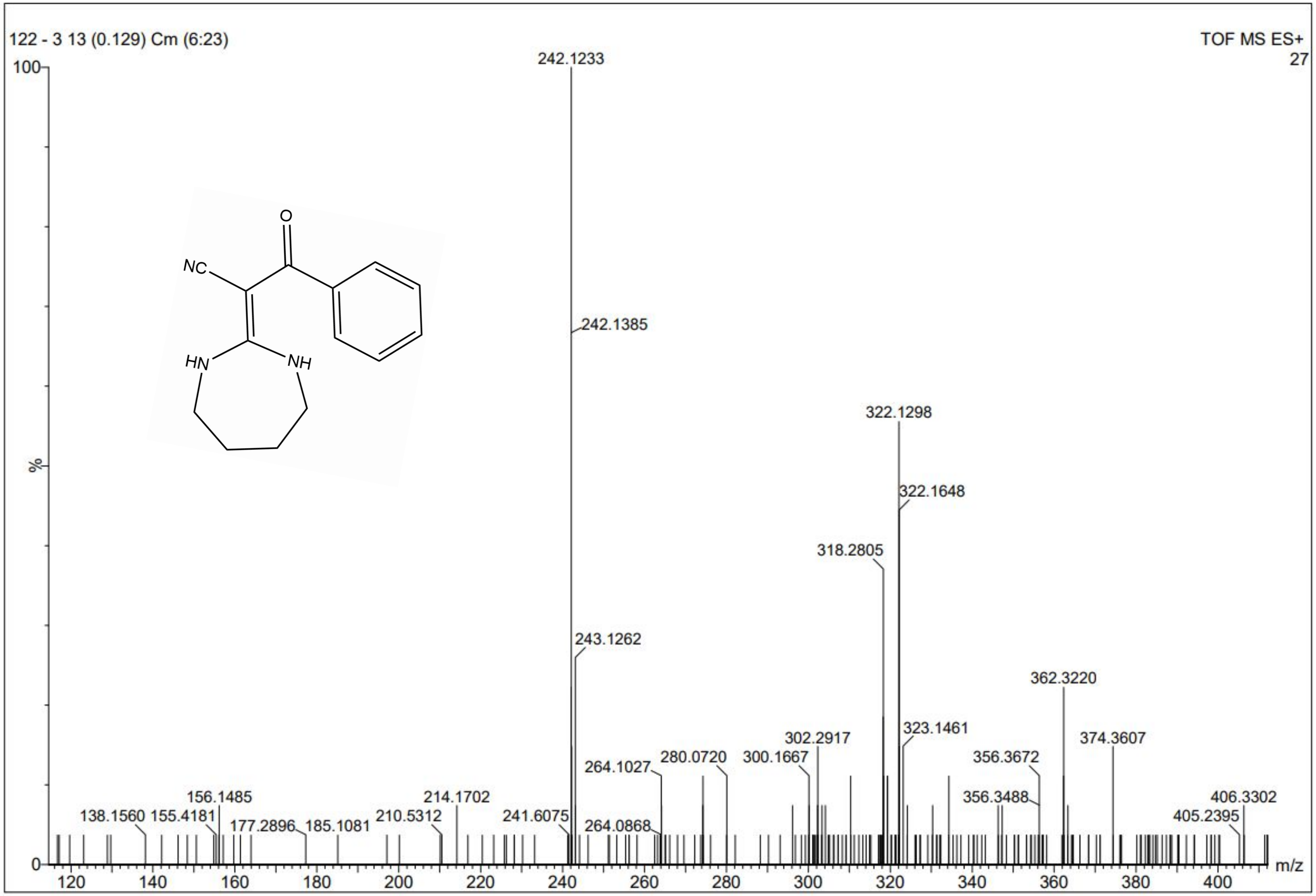

Figure S31. IR spectrum of 2-(1,3-diazepan-2-ylidene)-3-oxo-3-phenylpropanenitrile (**14**).

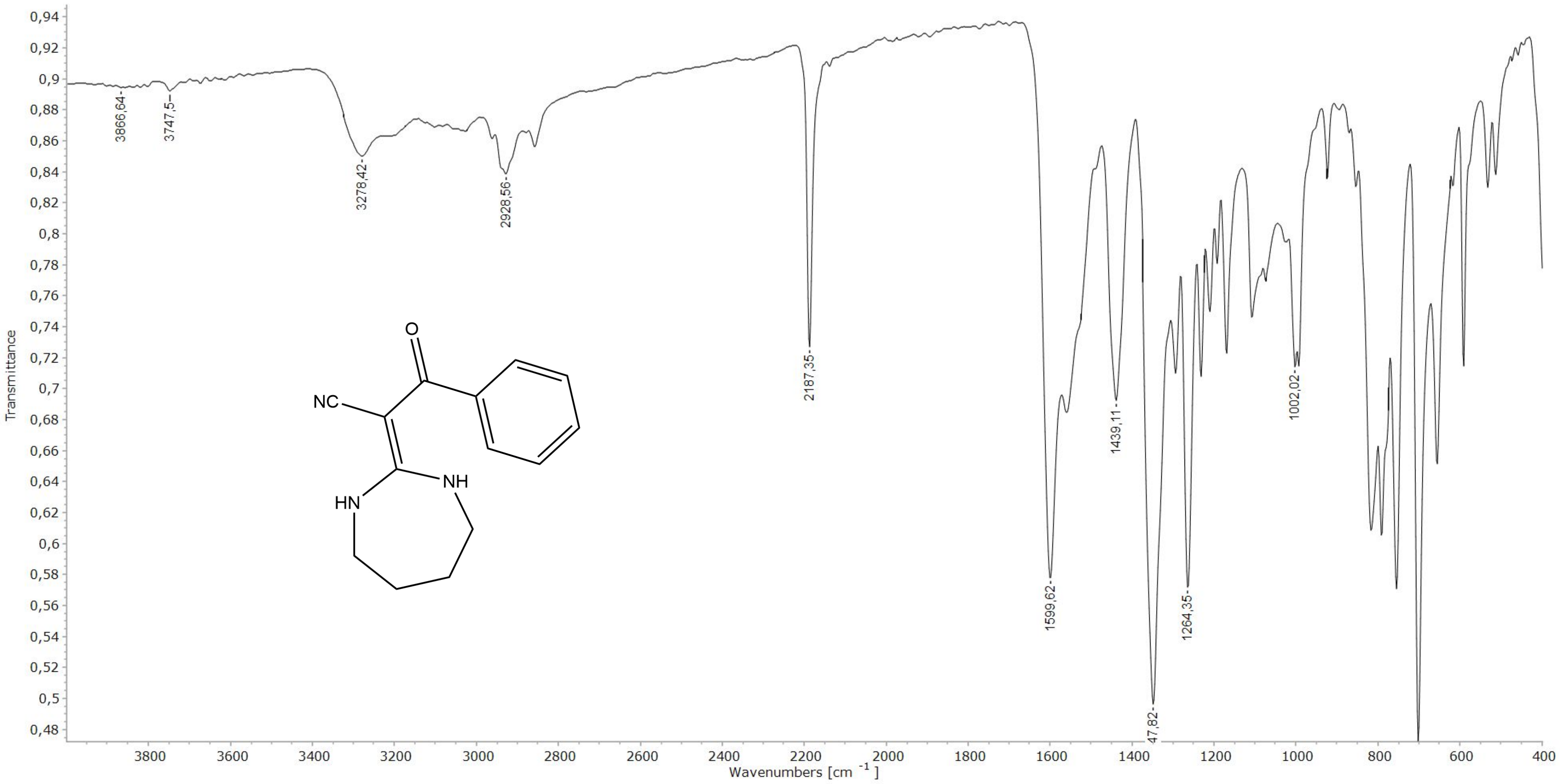

Figure S32. Cytotoxic activity of synthesized compounds **10-14** against MDA-MB-231 (A), A549 (B), TOV-21G (C) and WI-26VA4 (D) cell lines.

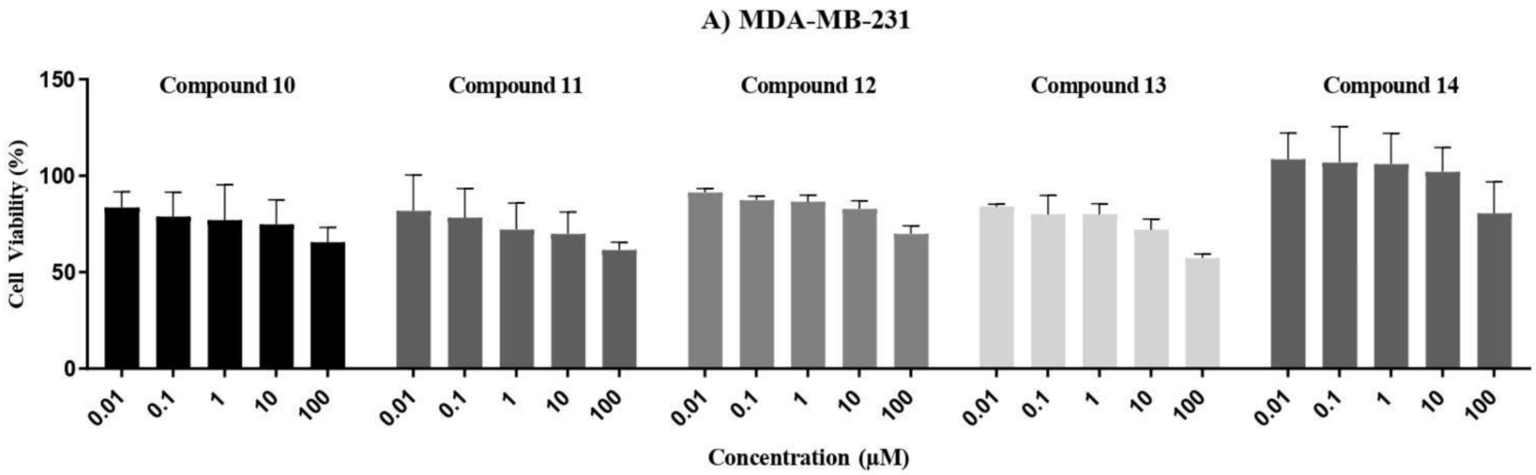
A) MDA-MB-231
Compound 10
Compound 11
Compound 12
Compound 13
Compound 14
Cell Viability (%)
150
100
50
0
0.01
0.1
1
10
100
Concentration (μM)

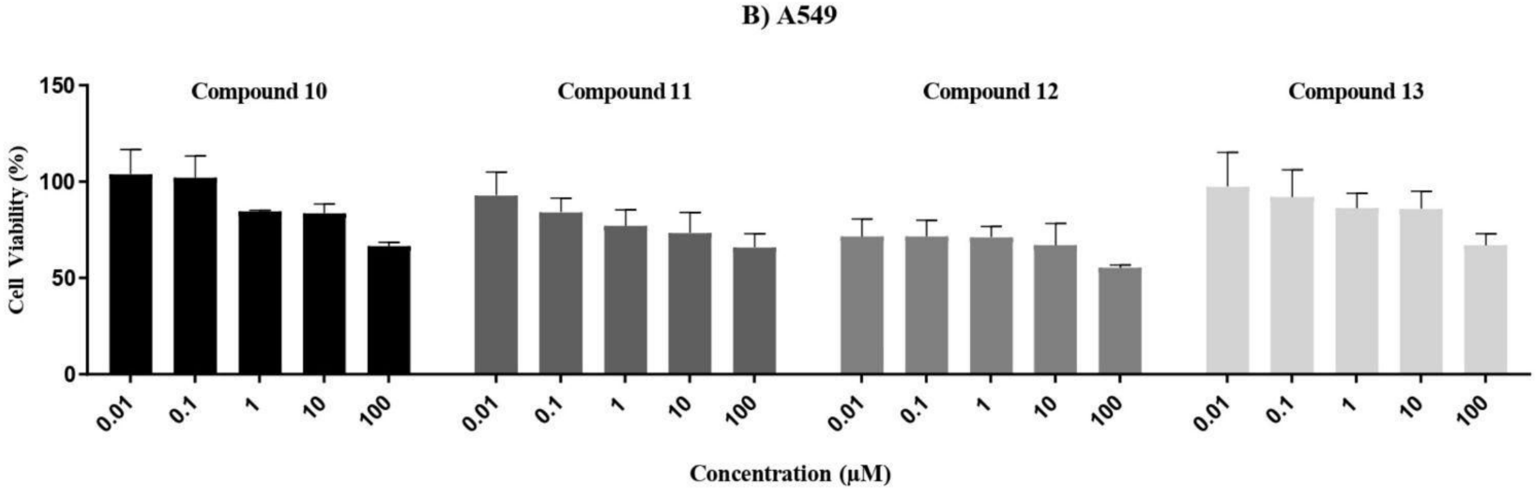
B) A549
Compound 10
Compound 11
Compound 12
Compound 13
Cell Viability (%)
150
100
50
0
0.01
0.1
1
10
100
Concentration (μM)

C) TOV-21G

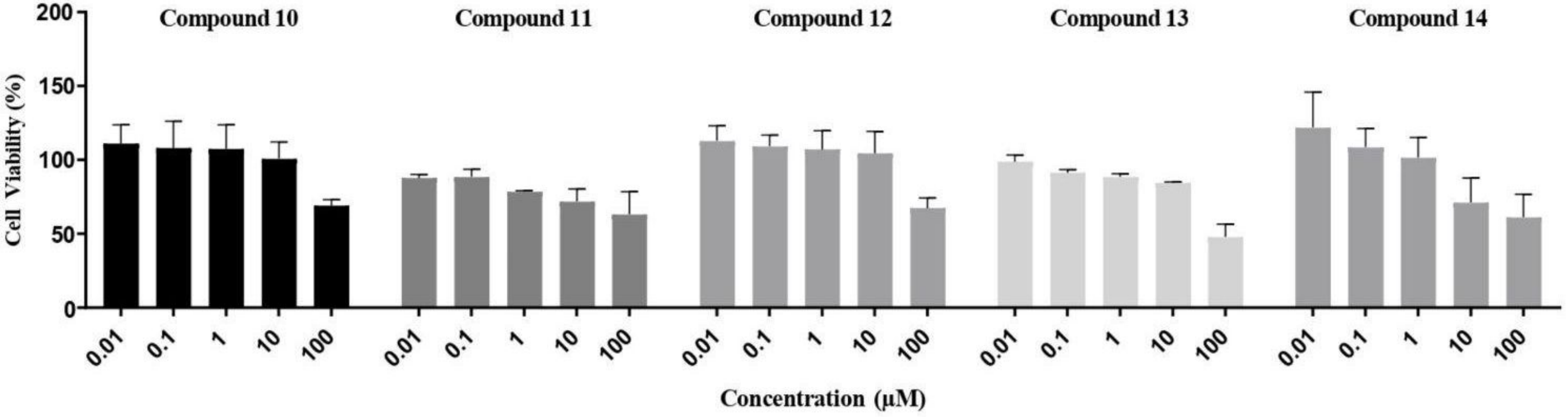

D) WI-26 VA4

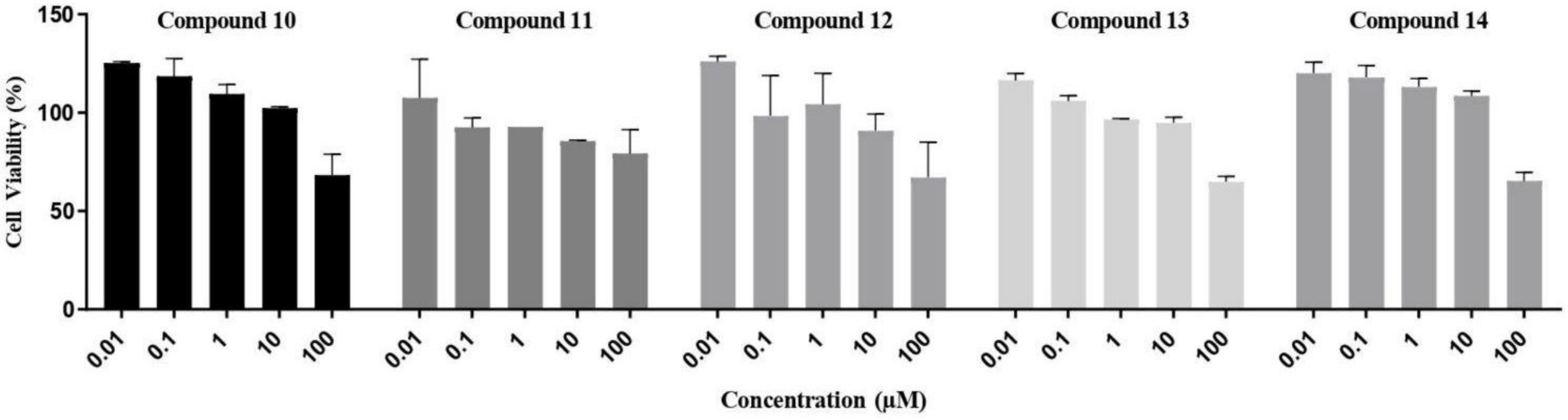

Figure S33. Dose-response graphs of doxorubicin on cell viability. Determination of half maximal inhibitory concentration (IC_{50}) against MDA-MB-231 (A), A549 (B), TOV-21G (C) and WI-26VA4 (D) cell lines.

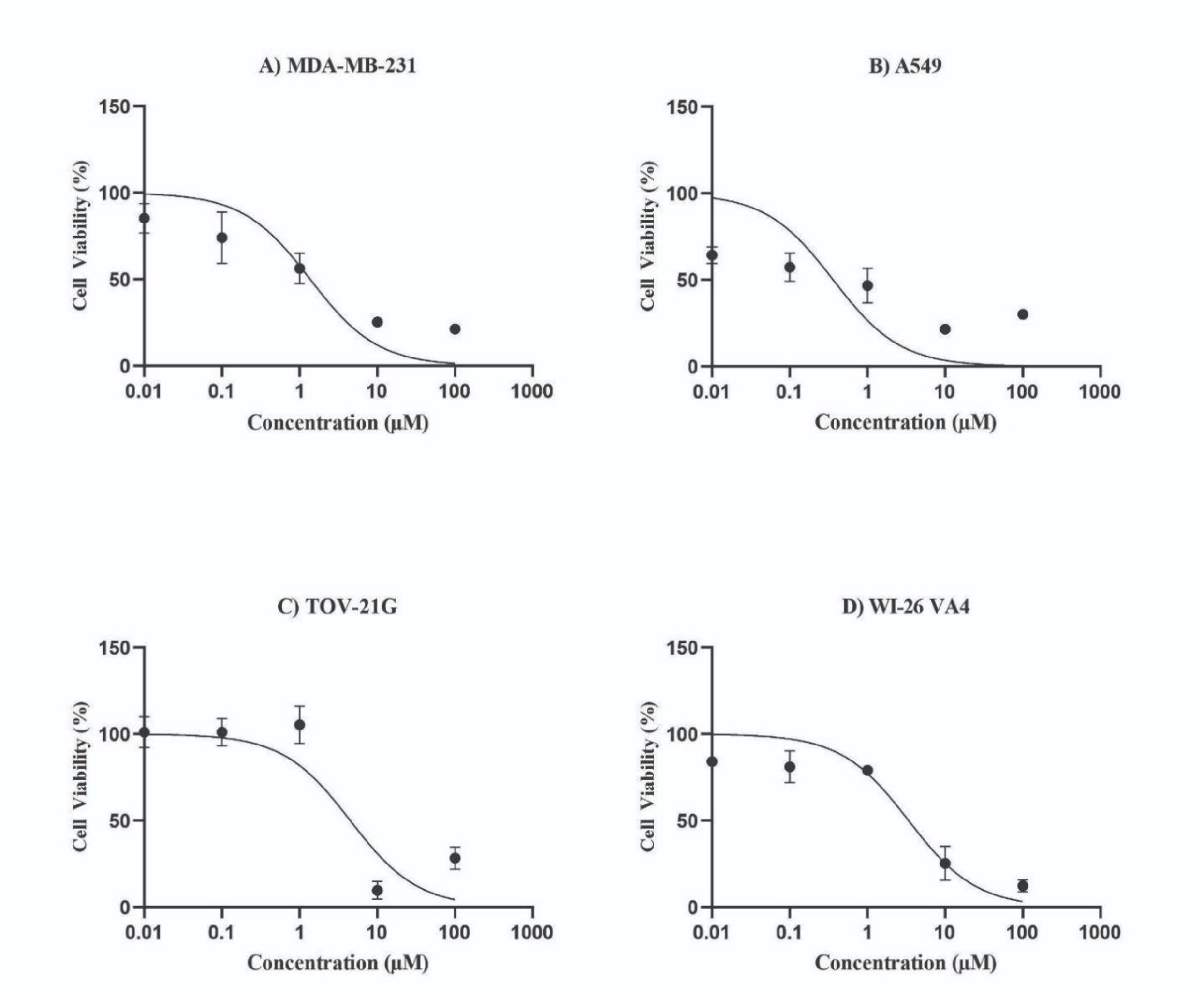

Supplement: Supplementary file 1 [file ao5c07989_si_001.pdf]
